# Uniting 4D Printing and Melt Electrowriting for the Enhancement of Regenerative Small Diameter Vascular Grafts

**DOI:** 10.1002/adhm.202502380

**Published:** 2025-08-07

**Authors:** Max von Witzleben, Akvilė Gasiūnaitė, Marlene Ihle, Ashwini Rahul Akkineni, Kathleen Schütz, Tilman Ahlfeld, Michael Gelinsky, Anja Lode, Sarah Duin

**Affiliations:** ^1^ Technische Universität Dresden University Hospital Carl Gustav Carus and Faculty of Medicine Centre for Translational Bone, Joint and Soft Tissue Research Fetscherstraße 74 01307 Dresden Germany

**Keywords:** 4D printing, additive manufacturing, alginate, extrusion 3D printing, melt electrowriting, methylcellulose, polycaprolactone

## Abstract

The development of mechanically robust, cell‐instructive, and seweable small‐diameter (≤ Ø 6 mm) tubular scaffolds remain a major challenge in vascular tissue engineering. Here, a hybrid biofabrication strategy is presented that combines 4D printing of alginate‐methylcellulose (AlgMC) hydrogels with melt electrowritten (MEW) poly(ε‐caprolactone) (PCL) reinforcement to produce tubular constructs with programmable shape‐morphing capacity. The MEW fiber meshes significantly improve mechanical integrity, enabling suturing and perfusion, while preserving the anisotropic swelling behavior required for morphogenesis. Scaffold functionalization using human blood‐derived protein coatings — such as fresh frozen plasma, platelet lysate, and fibrinogen — markedly enhances cellular adhesion and fibroblast proliferation without compromising structural transformation. Biological evaluation using mono and co‐cultures of fibroblasts, endothelial cells (HUVEC), and vascular smooth muscle cells (vSMC) reveals the formation of organized bi‐layers and phenotype‐specific cell morphologies on AlgMC/PCL composites. Notably, a confluent endothelial layer promotes contractile marker expression in vSMC, while vSMC support endothelial coverage in the absence of a growth‐arrested fibroblast feeder layer, indicating reciprocal stabilization. While further optimization is needed to meet the demands of small‐diameter vascular grafts fully, the presented system offers a versatile and promising platform for engineering soft tissue constructs that benefit from topographical guidance, spatially controlled adhesion, and adaptive geometry.

## Graphical abstract

A hybrid 4D printing strategy enables the fabrication of shape‐morphing, mechanically reinforced tubular constructs for vascular tissue engineering. By combining alginate‐methylcellulose hydrogels with melt electrowritten polycaprolactone fibers and protein‐based functionalization, this platform supports spatially organized co‐cultures of fibroblasts, endothelial, and smooth muscle cells, offering new opportunities for dynamic, cell‐instructive soft tissue scaffolds.

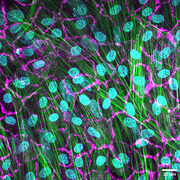

## 1 Introduction

The incidence of cardiovascular diseases is rising worldwide and is predicted to reach 22–23 million annually by 2030.^[^ ^1^ ^]^ In 2013, cardiovascular diseases were reported to be the leading cause of untimely death in all age groups above 40 years.^[^ ^2^ ^]^ These conditions mainly originate from atherosclerosis, which manifests in cholesterol plaque accumulation in the inner lining of the vessels, narrowing the passage and inducing an inflammatory response.^[^ ^3^ ^]^ This is often followed by thrombosis and complete blockage of the vessel, leading to a heart attack or stroke.^[^ ^4^ ^]^

The clinical standard of treatment is bypassing the blood supply through autologous grafts, thereby accepting donor site morbidity. However, pre‐existing diseased conditions and previous harvesting can exclude auto‐grafting.^[^ ^5^ ^]^ In this case, synthetic vascular grafts made of non‐degradable polymers are applied. They have proven to be suitable for the replacement of large vessels with a diameter wider than 6 mm and have been used clinically for more than half a century^[^ ^6^ ^]^ being the gold standard of artificial grafts to date.^[^ ^7^ ^]^ However, smaller diameter synthetic grafts experience differences in blood flow dynamics (high pressure) and often struggle with graft occlusion due to thrombosis since the platelets favor adhering to the hydrophobic polymer surface.^[^ ^8^ ^]^ In addition, they bear the risks of causing a foreign body reaction, infection or calcification, and they are never fully integrated due to their non‐biological composition, the rate of colonization by cells is low.^[^ ^5^, ^9^, ^10^ ^]^

Therefore, research aims to mimic the native properties of vasculature more closely. Improved synthetic grafts should have a cell‐friendly surface to enable colonization, since the coverage with endothelial cells would prevent platelet adhesion and thrombosis.^[^ ^8^ ^]^ This can be achieved, among others, by using biopolymers as scaffold materials and/or coatings to imitate the natural microenvironment.^[^ ^11^ ^]^ Tissue‐engineered vascular grafts, in which endothelialization occurs in vitro prior to implantation, have a high potential to replace autologous grafts for small diameter vessel replacement therapy.^[^ ^5^, ^11^ ^]^ Further requirements to be met by such an artificial graft are appropriate mechanical properties – stability and flexibility at the same time – as the graft has to be sutured to the native vessels, withstand the perfusion with blood without bursting, and follow the geometry of the organ.^[^ ^12^, ^13^ ^]^ Finally, biodegradability in a suitable time period would allow for full anatomical and functional recovery.^[^ ^14^ ^]^ Based on these considerations, the choice of biomaterial(s) and manufacturing method(s) for generating tubular structures of desired size as well as the in vitro cell colonization approach, are decisive aspects. Tailoring a material system, necessary to meet the various requirements for an artificial graft, can be realized by combining different biomaterials, by uniting different manufacturing techniques, and by functionalization with biological factors.

4D printing, a relatively recent advancement in manufacturing, builds upon 3D printing technology by incorporating time‐dependent transformations in printed objects.^[^ ^15^, ^16^ ^]^ It is considered an auspicious cutting‐edge technique for biomedical applications that is, among others, suitable for the fabrication of self‐sustainable tubular scaffolds for vascular grafts and stents.^[^ ^17^ ^]^ Recently, a biopolymer hydrogel blend of 3 wt% alginate (Alg) and 9 wt% methylcellulose (MC), developed for 3D extrusion bioprinting,^[^ ^18^ ^]^ has been reported as an applicable material for 4D printing: Lai et al. printed AlgMC into patterned, flat structures which consisted of hydrogel stripes on top of a continuous base layer. After drying, shape‐morphing of these hydrogel sheets into complex 3D structures was induced by rehydration and crosslinking via Ca^2+^ ions, which is based on the anisotropic stiffness and swelling behavior of the patterned hydrogel structures.^[^ ^19^ ^]^ Lai et al. reported that the direction of the shape‐morphing could be controlled by the alignment of the hydrogel stripes to achieve helical or tubular structures rolled at different angles.^[^ ^19^ ^]^

Self‐generating tubular structures are a promising approach for the generation of tissue‐engineered vascular grafts, especially if they could be tailored to a diameter smaller than 6 mm and reinforced mechanically. However, most hydrogels, when used independently, lack sufficient mechanical strength for their intended applications. An applicable method for reinforcement of a hydrogel is the use of synthetic (micro‐)fibers possessing high mechanical stability.^[^ ^20^, ^21^ ^]^ The advanced high‐resolution additive manufacturing technique of melt electrowriting (MEW) can generate microfibers out of polymer melts – the most prominent material used for MEW is polycaprolactone (PCL) – and deposit them with defined alignment to create an ordered pattern with high precision.^[^ ^22^, ^23^ ^]^ Thus, hierarchical meshes with specified fiber diameters and controlled porosity can be engineered to modulate the stiffness and strength of hydrogel‐mesh composites.^[^ ^24^, ^25^ ^]^ Herein it was studied whether the combination of 4D printing of a biopolymer‐based hydrogel construct with MEW of a fiber mesh enables the fabrication of small diameter vascular grafts with adequate mechanical stability and surface properties allowing cell colonization.

More precisely, it was aimed to develop a multi‐step fabrication process that combines 3D extrusion printing and MEW for the integration of PCL fiber meshes into a 4D printing process of an AlgMC‐based hydrogel. Process design and parameters were established that enable the generation of fiber‐reinforced hydrogel tubes with small diameter (≤ 6 mm) which are mechanically stable and could facilitate cell colonization. The mechanical properties of the fiber‐reinforced hydrogel tubes were characterized by tensile test, by analyzing their principal suitability for suture and perfusion. Their cell‐supportive properties were assessed by cell colonization of the composite structures. A strategy for an efficient endothelialization of the AlgMC‐PCL scaffolds was developed by protein functionalization and, finally, conditions for a bilayered co‐culture of smooth muscle cells and endothelial cells on the composites were established.

## 2 Results

### 2.1 Establishment of the Fabrication Process of PCL Mesh‐Reinforced Hydrogel Tubes

#### 2.1.1 4D Printing of Hydrogels Based on Alginate‐Methylcellulose

**Figure 1:**
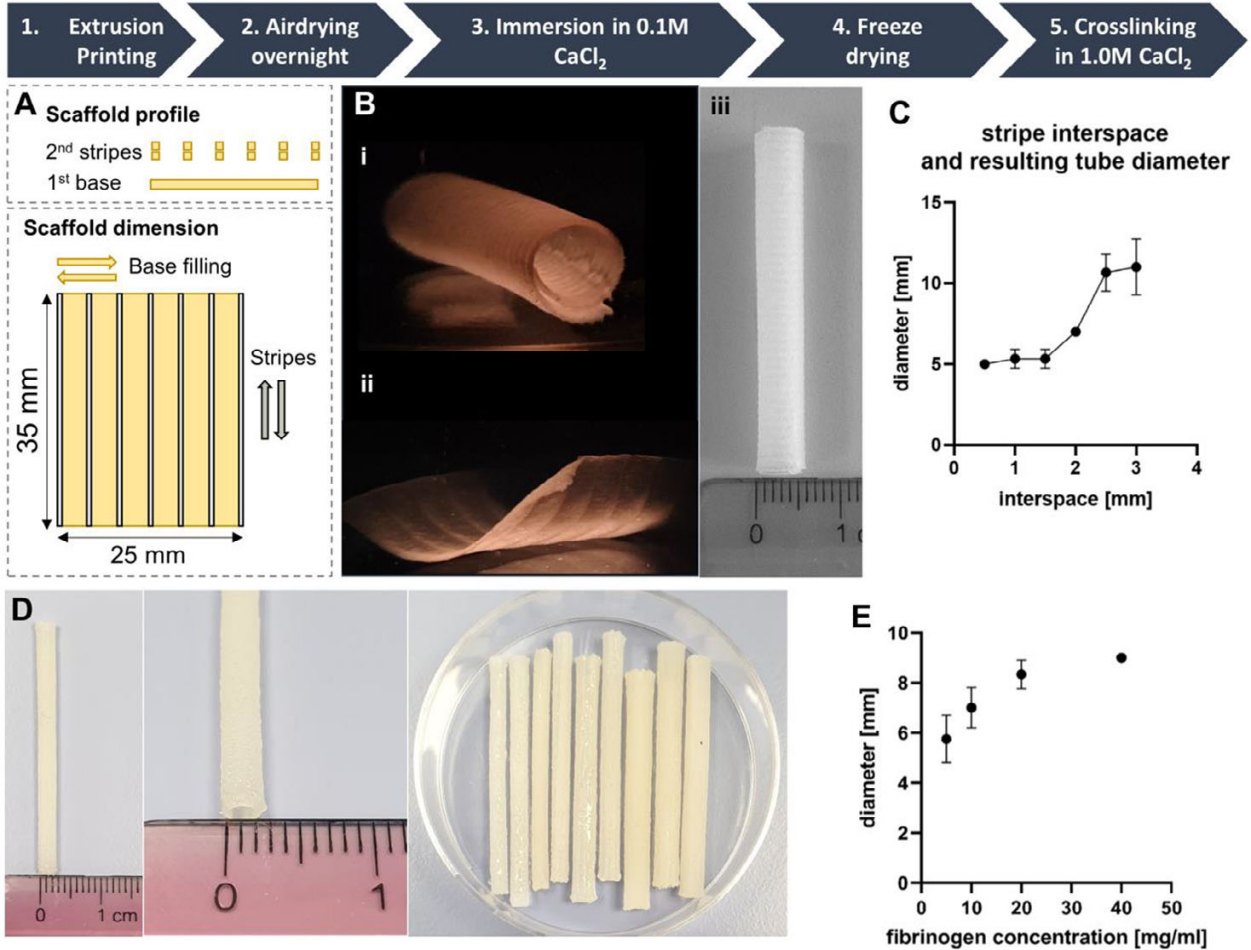
Depicted is the manufacturing process to obtain 4D printed tubular structures. The design A) was printed and then air‐dried overnight before being immersed in a CaCl_2_ solution, where the 4D shape morphing took place B). A stripe spacing of ≤ 3 mm resulted in tube formation (Bi) whereas a spacing of 4 mm did not allow shape morphing into tubes (Bii); a tube diameter of 5 mm was achieved by using a nozzle outlet diameter of 410 µm and a spacing of 0.7 mm (Biii). The tubular diameter depends on the stripe spacing, as demonstrated for structures printed with a 410 µm diameter nozzle C) and can be reduced by using a 200 µm diameter printing nozzle as well as a halved base area of the printed structure (35 mm × 12.5 mm) (D). Modification of AlgMC with fibrinogen affected the diameter of the formed tubes in a concentration‐dependent manner E).

Tubular scaffolds were fabricated via the following protocol, confirming previous findings^[^ ^19^ ^]^ (**Figure** **1**): An extrusion‐printed AlgMC hydrogel structure was dried overnight in air and then immersed in pre‐crosslinking solution (0.1 m calcium chloride, CaCl_2_) to induce shape morphing in the tubes (Video S1, Supporting Information). The formed tubes were lyophilized and finally crosslinked in 1 m CaCl_2_ for mechanical stability prior to use. This increases storability, offering the possibility of off‐the‐shelf usage for clinicians. It was found that the printing process and the printed scaffold geometry strongly influenced the resulting tube diameter. In general, the printed structure (35 mm × 25 mm) consisted of three layers with a dense base layer printed orthogonal to the top two layers of stripes (Figure 1A). Three factors were found to be influential on the shape‐morphing properties: first, the spacing of the strands; second, the outlet diameter of the nozzle used to print the hydrogel to define the width of the strands; and third, the dimensions of the base layer. With a nozzle outlet diameter of 410 µm, tube formation was achieved with a strand spacing of up to 3 mm; the smallest tube diameter of 5 mm was obtained at a spacing of 0.5–1.5 mm, while larger spacing increased the tube diameter (Figure 1B,C). The reduction of the outlet diameter of the nozzle to 200 µm resulted in thinner stripes and layers. With a spacing of 0.7 mm between the strands and a halved base area of the printed structure (35 mm × 12.5 mm), tubes with an inner diameter of 2–3 mm were formed (Figure 1D). Since AlgMC lacks cell adhesion sites, it was investigated whether AlgMC inks supplemented with cell‐supportive components are still capable of shape morphing into tubular structures. A modification with human fresh frozen plasma (FFP), previously shown to significantly increase the functionality of the inks,^[^ ^26^ ^]^ did not allow the morphology switch in CaCl_2_ and thus, attempts to obtain tubular grafts were unsuccessful (data not shown). In contrast, the incorporation of fibrinogen into the hydrogel ink (5–40 mg ml^−1^) did not impair shape morphing of the printed structures. Interestingly, with rising fibrinogen concentration, the tube diameters increased; only with the lowest concentration of 5 mg ml^−1^ tube formation was comparable to AlgMC without fibrinogen (Figure 1E).

#### 2.1.2 Embedding of Melt Electrowritten Polycaprolactone Meshes

**Figure 2:**
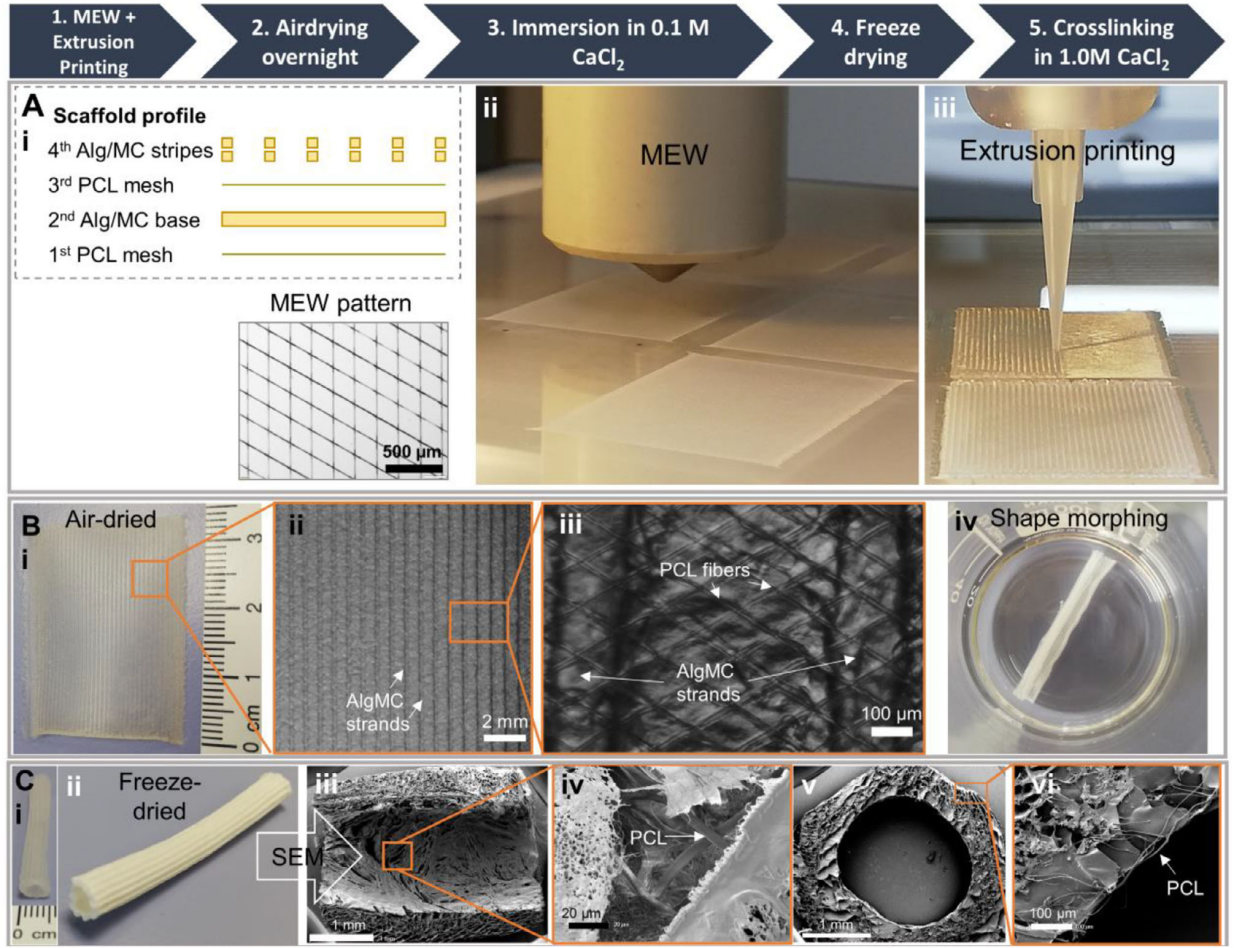
Depicted is the manufacturing process to obtain composite tubular structures. The alternating design between MEW and extrusion printing allows for a heterogeneous composition (Ai–iii). The flat air‐dried scaffolds demonstrate the hydrogel and the PCL printing pattern (Bi–iii). After immersion in CaCl_2_ (Biv), the shape morphing effect resulted in small diameter grafts (< 5 mm, Ci‐ii) with PCL microfibers sunk into the hydrogel, as indicated by (SEM analysis (Ciii–vi)).

Pure hydrogel tubes were rather fragile in handling, especially when re‐immersed in fluids. Therefore, it was investigated whether melt electrowritten polycaprolactone (PCL) meshes could be incorporated into the structures to enhance the mechanical properties of the obtained grafts. Different variants to combine MEW PCL meshes with the extrusion‐printed hydrogel structures were tested and the variant, which resulted in a stable construct throughout all process steps, is presented in **Figure** **2**: Two layers were added to the print design (Figure 2Ai), i.e., first a MEW PCL mesh was printed (Figure 2Aii), then the hydrogel base, then a second MEW PCL mesh and finally the two hydrogel stripe layers (Figure 2Aiii). Each MEW PCL mesh consisted of four layers, with a 60° layer‐to‐layer orientation, a fiber diameter of 7 ± 1.5 µm, and a fiber spacing of 100 µm. Several other mesh designs with different angles, number of layers, and fiber distances were tested, but these modifications either increased the tube diameter or inhibited the tube formation completely (Figure S1, Supporting Information). By using the optimal design, the hydrogel and the MEW PCL mesh were merged, and shape morphing into tubes occurred (Figure 2B). The resulting tube formation was not affected by this MEW fiber configuration, and comparable small diameter tubes of 5 mm were achieved (Figure 2Ci+Cii). Images taken with a scanning electron microscope (SEM) highlight the integration of the PCL fibers into the hydrogel, hence preventing a delamination (Figure 2Cii‐vi).

Through this newly established manufacturing approach, mesh‐reinforced hydrogel tubes with small diameters (≈ 5 mm) can be generated by combining 3D extrusion‐based printing of AlgMC and MEW of PCL in one fabrication process. Both the design of the AlgMC printed structure and the MEW mesh have a significant impact on the shape‐morphing effect into tubular structures (Figure 1+ S1, Supporting Information).

### 2.2 Characterization of MEW PCL Mesh‐Reinforced Hydrogel Tubes

#### 2.2.1 Mechanical Analysis

**Figure 3:**
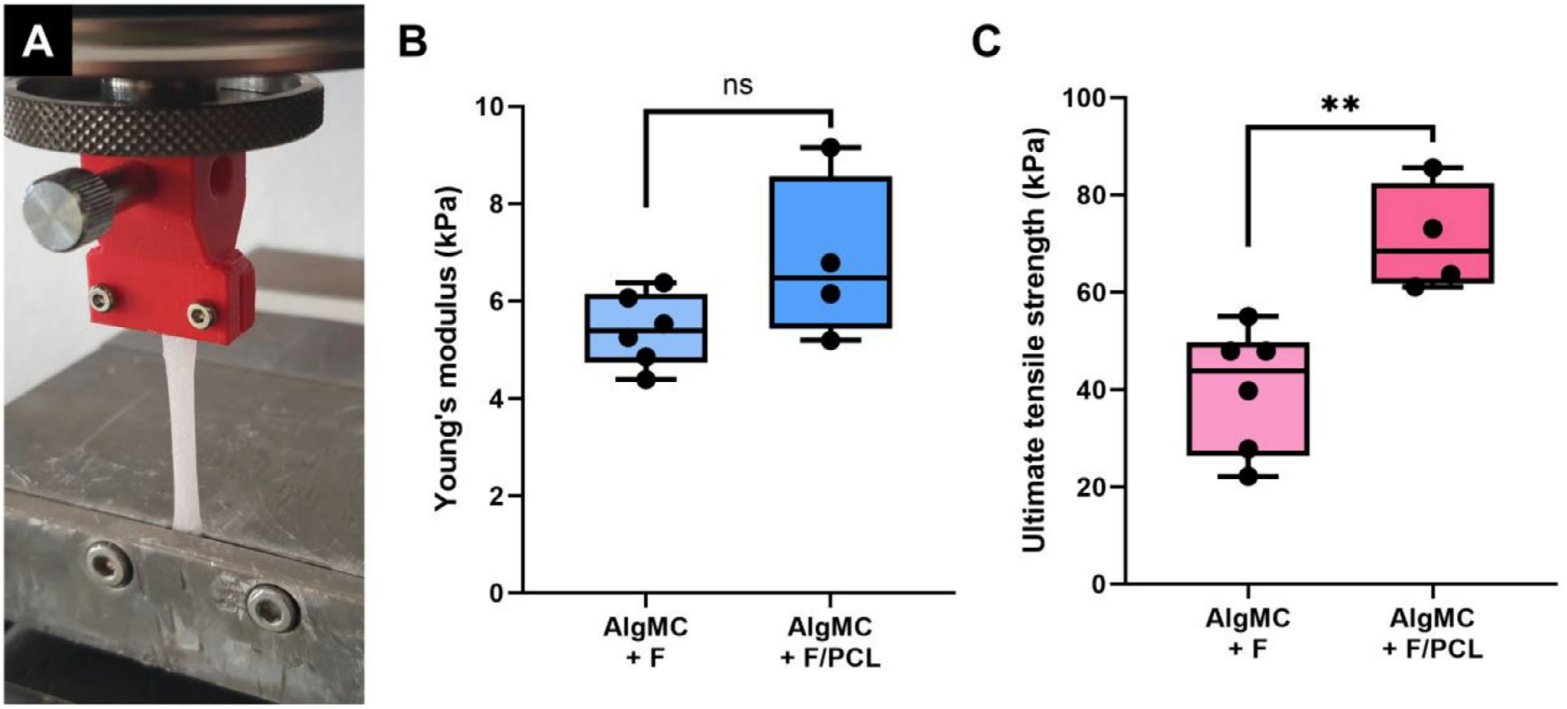
The mechanical properties of the tubes were tested with tensile tests A). The Young's modulus B) and the ultimate tensile strength (UTS) C) were analyzed depending on the reinforcement of fibrinogen (F; 5 mg ml^−1^) modified AlgMC with MEW PCL meshes. (ns  =  not significant; ^**^p < 0.01).

Tensile tests were performed to assess whether the MEW mesh‐reinforcement has an impact on the mechanical properties of the 4D printed hydrogel structures (**Figure** **3**). The incorporation of the MEW PCL meshes did not significantly alter the Young's modulus but significantly increased the ultimate tensile strength (UTS). Consequently, the PCL fiber meshes did not modify the flexibility of the constructs but increased the maximum applicable force.

#### 2.2.2 Clinical handling and perfusion

**Figure 4:**
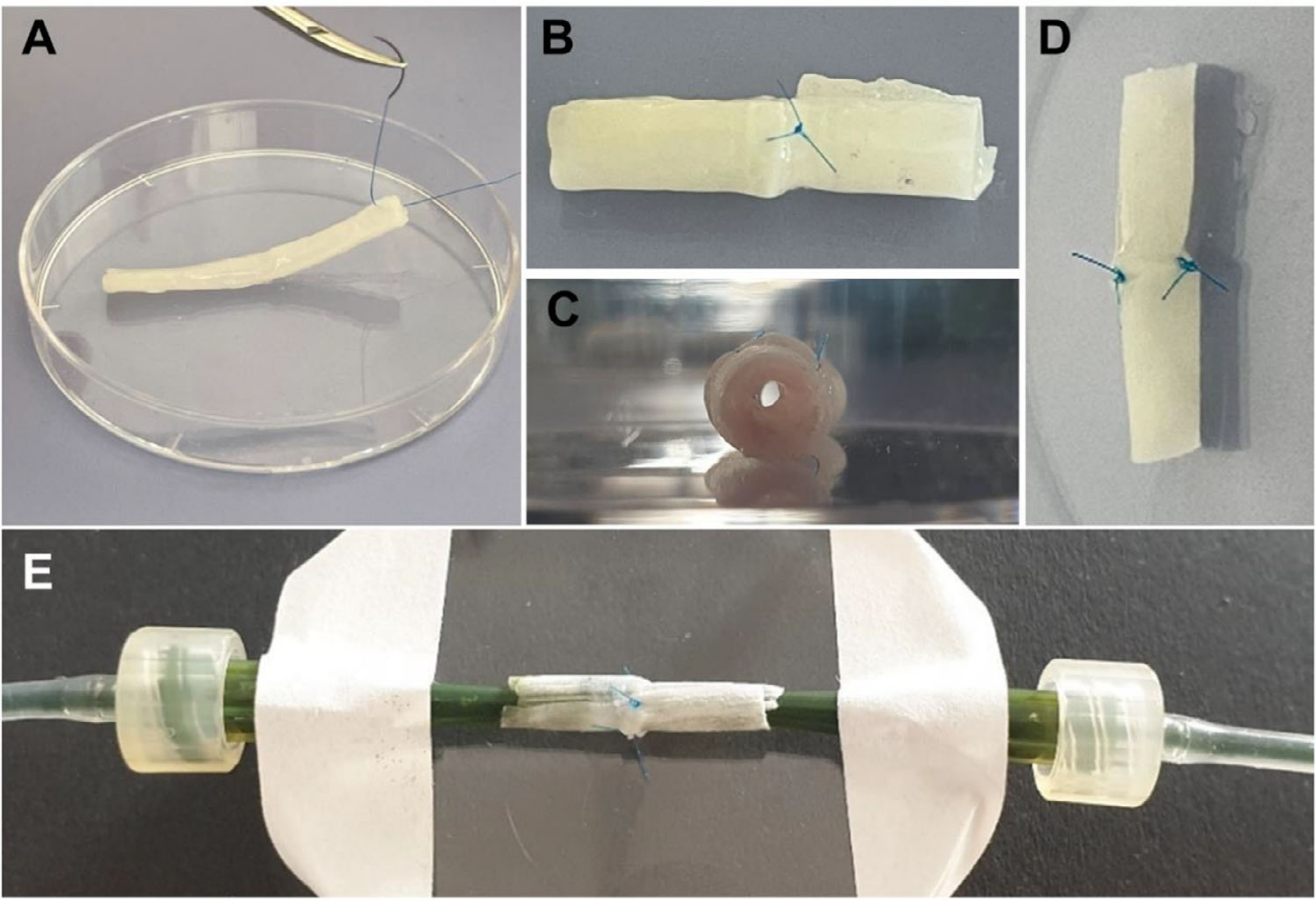
The general applicability of the tubular scaffolds as vascular grafts was tested successfully with sewing A–D) and perfusion tests E).

The clinical manageability of the constructs is critical for the possible application and thus, the ability of the composites to allow for suturing by plastic surgeons and perfusion was tested (**Figure** **4**). Preliminary sewing tests with pure hydrogel tubes were not successful, and the needle could not be inserted without losing the integrity of the scaffolds (data not shown). With the integration of MEW PCL meshes, the mechanical behavior changed, as suggested by the UTS (Figure 3C). The needle was inserted without disrupting the scaffold, and a volunteer surgeon successfully sutured the incised sections using a surgical suturing kit, with the lumen remaining patent throughout the procedure (Figure 4A–D). Perfusion tests with water containing a food colorant showed no signs of leakage independent of flow velocity (Video S2, Supporting Information). Even the sutured tubes showed no leakage (Figure 4E; Video S3, Supporting Information), underlining the potential of this MEW‐integrated 4D printing approach. However, proof‐of principle investigations used perfusion speeds of 1–1.5 cm s^−1^ generating pressures (0.4–0.8 ×10^−3^ mmHg) far below the required 90–120 mmHg for arteries and 5–15 mmHg for veins.^[^ ^27^, ^28^ ^]^

#### 2.2.3 Cytocompatibility

**Figure 5:**
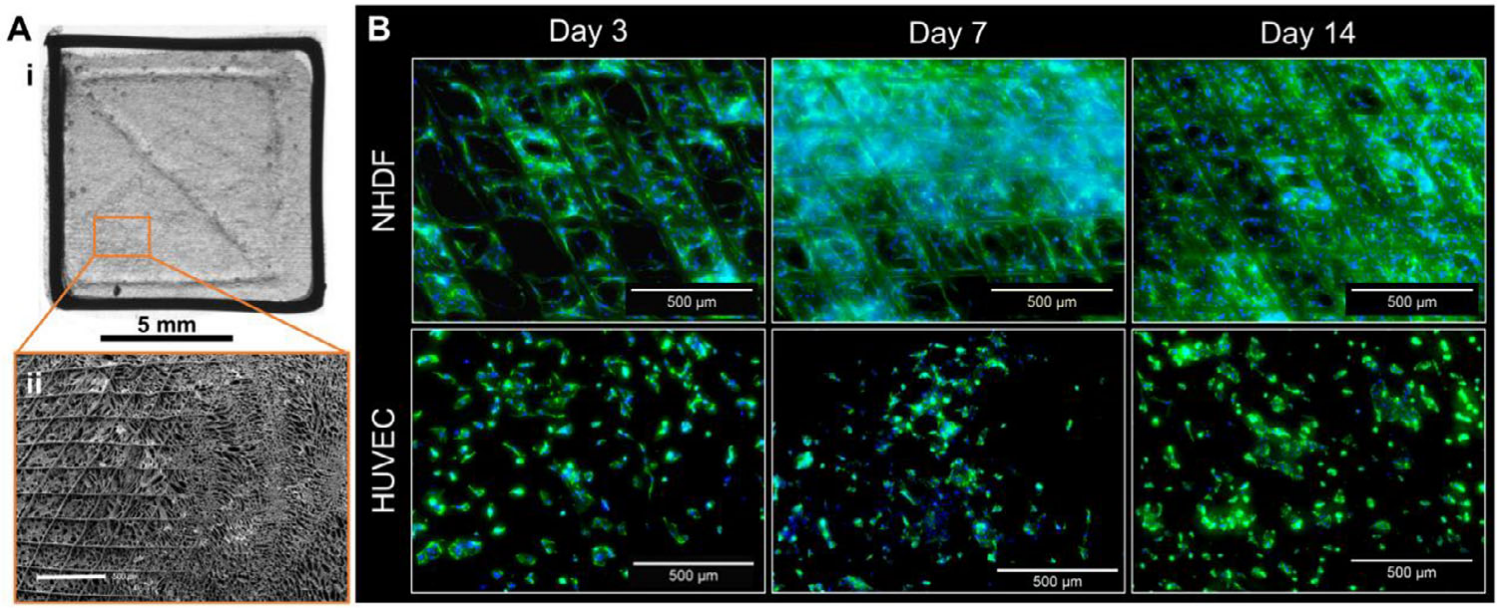
Flat composite scaffolds (Ai) were used for cytocompatibility tests; SEM analysis confirmed the incorporation of the MEW PCL mesh into the AlgMC+F hydrogel (Aii; scale bar = 500 µm). NHDF and HUVEC (B) were seeded on scaffolds and cultured for up to 14 days. The nuclei were stained with DAPI (blue) and the cytoskeletons with Phalloidin 488 (green); scale bars = 500 µm.

Initially, attachment to the composite material as well as proliferation of fibroblasts and endothelial cells were investigated to assess the ability of the grafts to support cell colonization (**Figure** **5**). Flat composite scaffolds (Figure 5A) were prepared to simplify the cell‐seeding process and the fluorescence‐microscopic analysis. Therefore, a sterile hydrogel base of AlgMC supplemented with 5 mg mL^−1^ fibrinogen was printed before manually adding a sterile MEW PCL mesh on top. Scaffolds were air‐dried overnight in a sterile cabinet, crosslinked, and then seeded either with Normal Human Dermal Fibroblasts (NHDF) or with Human Umbilical Vein Endothelial Cells (HUVEC). After 3, 7, and 14 days of cultivation, cell colonization of the scaffolds was examined using fluorescence microscopy after staining of cell nuclei and actin cytoskeletons. NHDF specifically attached to the PCL fibers and migrated along them; over the cultivation time, the cell density increased to finally cover the scaffold area and build a nearly closed cell layer (Figure 5B). In the absence of the PCL mesh, a lower cell density and a less uniform cell coverage was observed on pure hydrogel scaffolds consisting of AlgMC+F (Figure S2, Supporting Information). HUVEC adhered to the composite scaffolds with lower efficiency than the NHDF and did not form a closed cell layer within the culture period (Figure 5B).

To summarize insights into the mechanical and biocompatibility characterization, MEW PCL mesh reinforcement of the AlgMC‐based hydrogel tubes led to a significantly increased ultimate tensile strength and an improved fibroblast attachment. In addition, it allowed suturing of the tubes as well as their perfusion. The presence of the MEW PCL mesh is crucial for the success of a shape‐morphing 4D printing approach to meet clinical requirements.

### 2.3 Endothelialization

#### 2.3.1 Protein Functionalization After Fabrication for Optimized Cell Colonization

We first supplemented the AlgMC hydrogel with cell‐supporting additives before printing to enhance HUVEC colonization, but this approach impaired – or, at fibrinogen concentrations higher than 5 mg mL^−1^, completely prevented – tube morphing without improving cell growth. Consequently, we investigated post‐fabrication functionalization as a strategy to boost cell colonization while preserving tube formation and dimensions.

Composite scaffolds consisting of a printed AlgMC hydrogel base and a MEW PCL mesh were freeze‐dried after crosslinking and then rehydrated for 2 h with protein‐rich solutions to achieve protein coating/infiltration.

Initially, five different compositions were compared regarding their ability to support attachment of NHDF to the composite scaffolds: cell culture medium containing 10% fetal calf serum (FCS), 100% FCS, 100% FFP), collagen type I (human, 150 µg mL^−1^), and its derivative gelatin (bovine, 0.1%). The evaluation of cell adhesion one day after seeding with NHDF revealed that both cell culture medium and pure FCS did not support cell attachment and spreading, whereas FFP had a strikingly positive influence on cell adhesion and spreading on the surface of the scaffold with large confluent areas (Figure S3, Supporting Information). The collagen seemed to primarily coat the PCL fibers and encourage the cells to spread along them; similarly, the cells seeded on the gelatin‐coated scaffolds preferentially adhered to the PCL fiber mesh with slightly reduced viability (Figure S3, Supporting Information).

**Figure 6:**
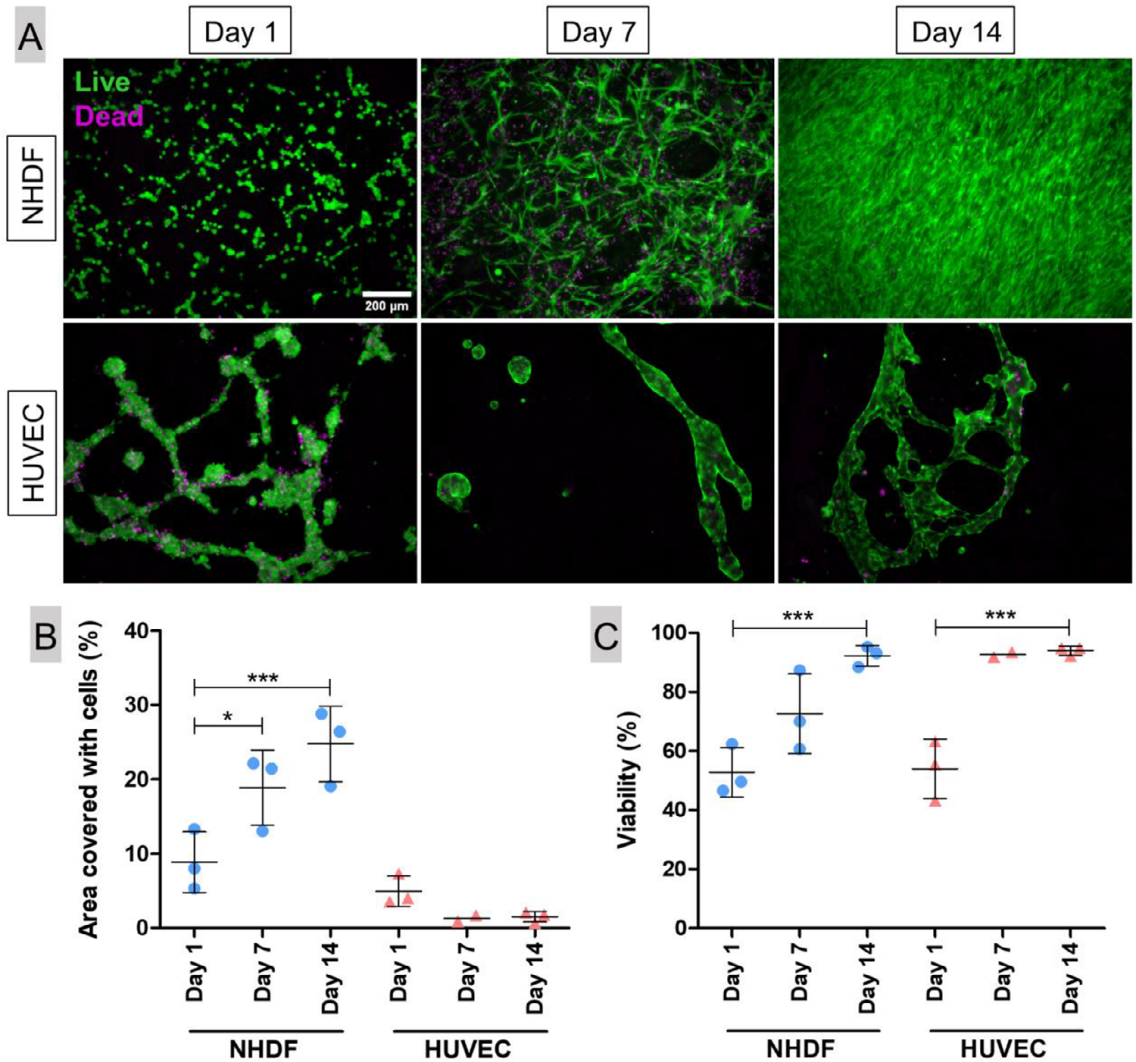
Adhesion, proliferation, and viability of NHDF (1 × 10^5^ NHDF/scaffold) or HUVEC (1.5 × 10^5^/scaffold) seeded on flat composite scaffolds of AlgMC hydrogel and PCL mesh, functionalized by rehydration in FFP. Exemplary images of simultaneous staining of live (green) and dead (magenta) cells was performed after 1, 7, and 14 days of cultivation: A) exemplary fluorescence‐microscopic images, scale bar  =  200 µm for all. The percentage of the whole scaffold area within the frame covered with cells B) and proportion of viable cells C) was determined by analysis of two to three scaffolds (n  =  2‐3; mean ± SD). One‐way ANOVA, Bonferroni's multiple comparison post‐hoc test. Significant differences represented as ^***^p<0.0001, ^*^p<0.05.

Functionalization with FFP was further assessed regarding its ability to support cell proliferation (**Figure** **6**). NHDF or HUVEC were seeded onto FFP‐functionalized AlgMC/PCL composite scaffolds, and the cell density on the scaffolds was observed over 14 days of cultivation. Overall, the NHDF exhibited proliferation indicated by an increased scaffold coverage over time (Figure 6A,B) and a high viability, which increased over the cultivation period (Figure 6C). Similar results were obtained when platelet lysate (PL), another type of human blood derivative rich in growth factors and fibrin, was used instead of FFP (Figure S4, Supporting Information). In contrast to NHDF, HUVEC seeded onto FFP‐functionalized AlgMC/PCL composite scaffolds showed an uneven distribution over the scaffold surface and an insufficient cell adhesion since many cells stayed round, collected in cell clusters; they formed a tubular network among themselves rather than spreading into a monolayer (Figure 6A). Despite an increased viability over the cultivation time, the area of coverage was even reduced after 7 and 14 days of culture compared to day 1 (Figure 6B,C). The inefficient colonization with HUVEC was also observed for composite scaffolds functionalized with collagen and PL (Figure S5, Supporting Information).

#### 2.3.2 Endothelialization Supported by NHDF

**Figure 7:**
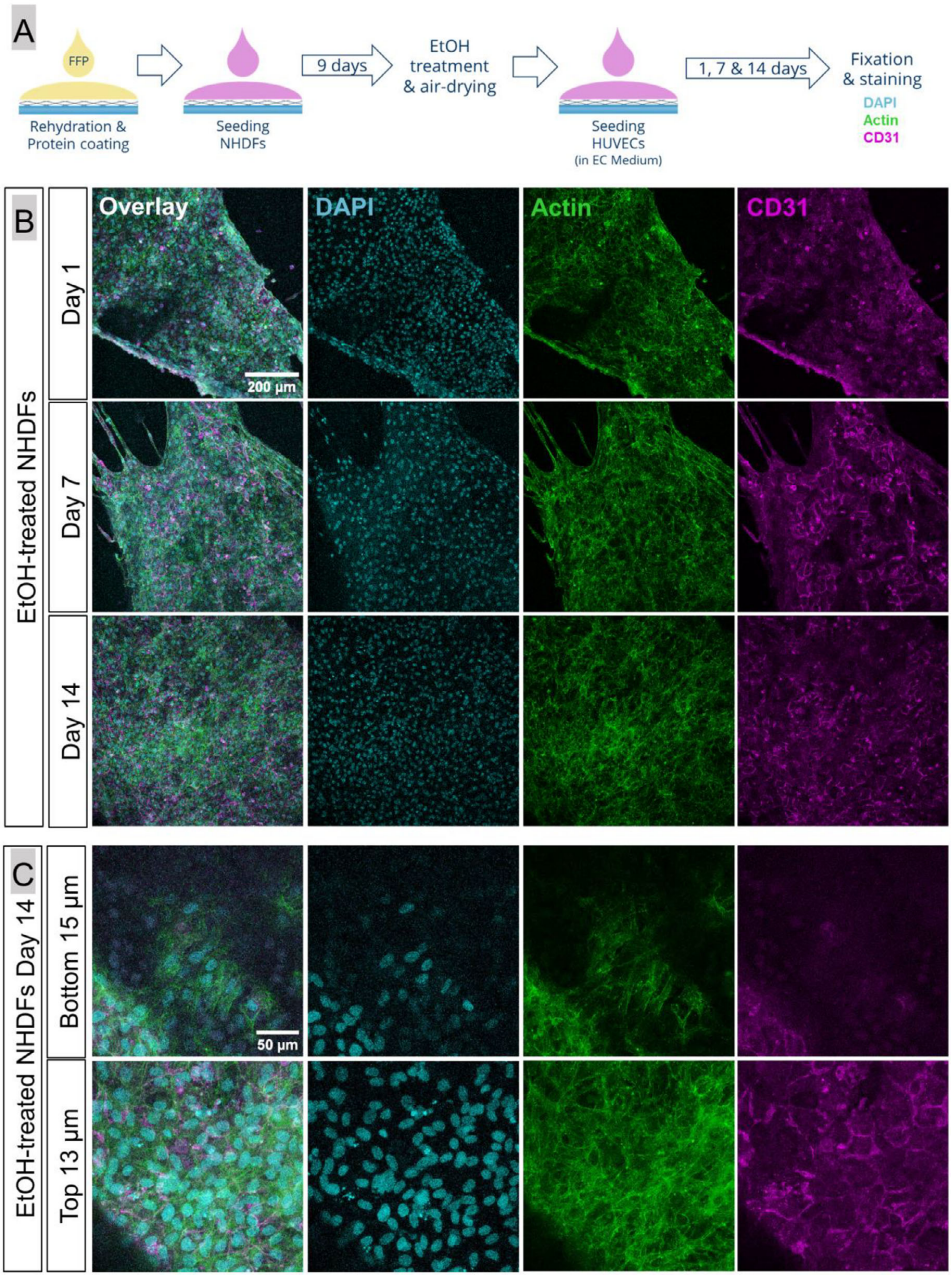
Formation of a HUVEC layer on scaffolds with an EtOH‐treated NHDF layer. The experimental setup is shown in A); 2.3 × 10^5^ HUVEC/scaffold were used for seeding. After 1, 7, and 14 days of cultivation in endothelial cell (EC) medium, samples were stained for cell nuclei with DAPI (blue), cytoskeletons with Phalloidin 488 (green), and the endothelial marker CD31 (magenta); scale bar  =  200 µm for all B). Confocal laser scanning microscopy over 28 µm revealed that the HUVEC stayed on top of the NHDF layer, indicated by the presence of the CD31 signal in the “Top 13 µm” and its nearly complete absence in the “Bottom 15 µm”; scale bar  =  50 µm for all C).

The inability to achieve efficient HUVEC adhesion and growth on the functionalized scaffolds indicated that an additional stimulus was required for these cells. Therefore, cell‐based support systems were investigated regarding their potential to stimulate the generation of a confluent HUVEC monolayer. A dense, widespread feeder layer of NHDF was established on FFP‐functionalized AlgMC/PCL composite scaffolds over a cultivation period of 10 days; thereafter, HUVEC were seeded on top and cultivated in their specific growth medium over 14 days (Figure S6A, Supporting Information). In a second approach, the NHDF were treated with ethanol (EtOH) to arrest the growth of the feeder layer at day 9 of the fibroblast culture and thus prevent overpopulation of the HUVEC by the quickly proliferating NHDF. After air‐drying of the EtOH‐treated samples, HUVEC were seeded and cultivated in their specific growth medium (**Figure** **7**A).

After seeding HUVEC on a live NHDF layer, they efficiently adhered and spread into a tightly packed layer over large areas within one day as indicated by CD31 staining. However, live NHDF continued to proliferate, and congregated cell masses appeared after 7 and 14 days of cultivation, reducing the expansion of HUVEC layer (Figure S6B, Supporting Information). After 14 days, the strongly proliferating NHDF led to the detachment of the cell multilayer/sheet (Figure S7). After seeding of HUVEC on EtOH‐treated NHDF layer, an efficient attachment and spreading of the endothelial cells was observed on day 1 as well. In contrast to the live NHDF layer, the EtOH‐treated NHDF layer could not only support the HUVEC layer formation, but also maintain it during further cultivation (Figure 7B; Figure S7, Supporting Information). The layered arrangement of NHDF and HUVEC was preserved throughout the 14 days of cultivation (Figure 7C).

Different strategies to achieve endothelialization were tested, and it was observed that while the functionalization of the AlgMC/PCL composite scaffolds with FFP strongly supported adhesion and proliferation of fibroblasts, endothelial cells were not able to efficiently adhere and proliferate. Nevertheless, after seeding HUVEC on top of a dense EtOH‐treated fibroblast layer, endothelial cells organized into a confluent and stable monolayer.

### 2.4 Bi‐Layered co‐Cultures of Smooth Muscle Cells and Endothelial Cells

**Figure 8:**
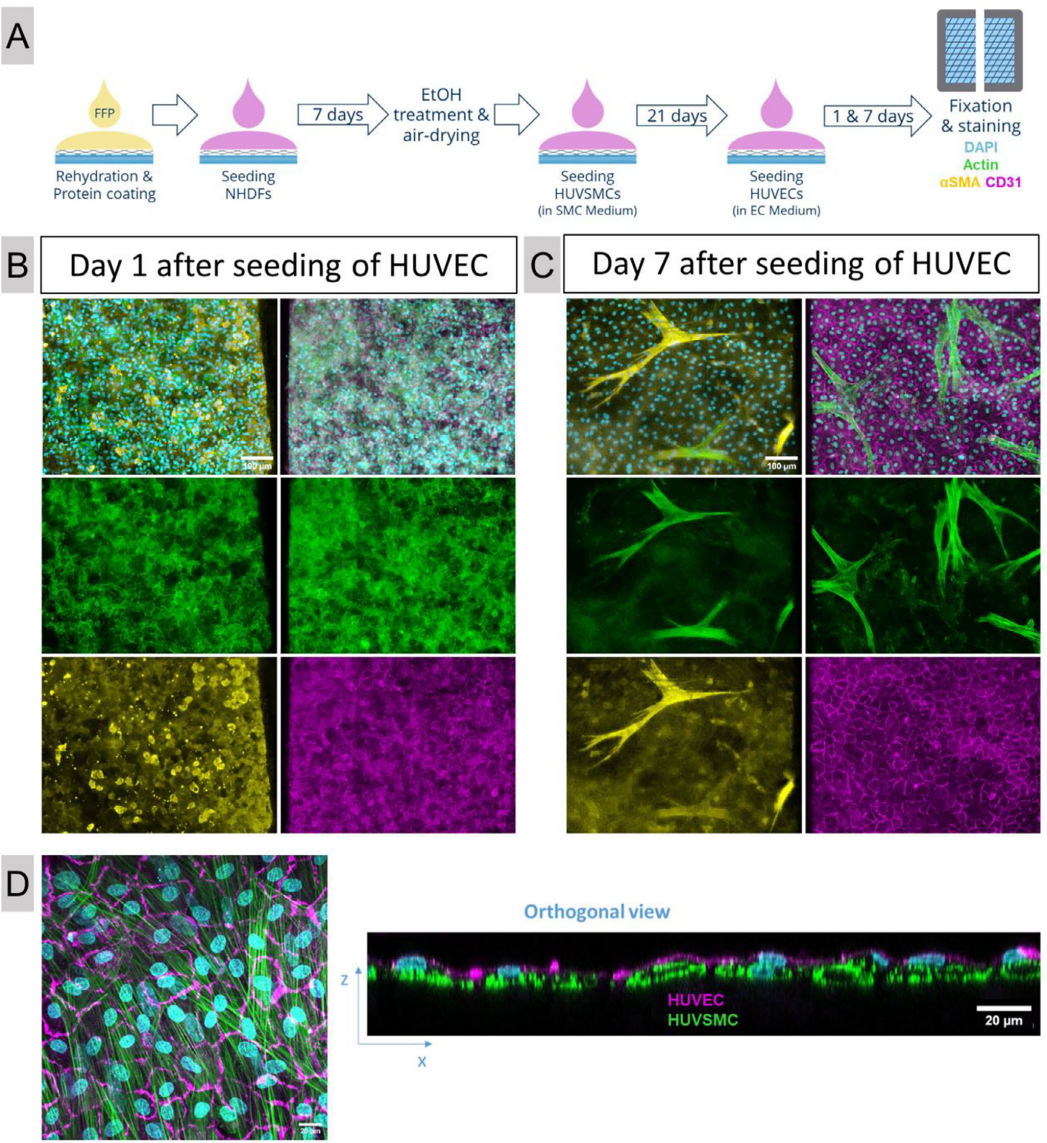
Formation of a bi‐layered co‐culture of HUVSMC and HUVEC on scaffolds with an EtOH‐treated NHDF layer. The experimental setup is shown in A); 1.5 × 10^5^ HUVSMC/scaffold and 2.3 × 10^5^ HUVEC/scaffold were used for seeding. After 1 B) and 7 C) days of cultivation after HUVEC seeding, samples were fixed and cut into half. One half was stained for cell nuclei with DAPI (blue), cytoskeletons with Phalloidin 488 (green) and the smooth muscle cell marker αSMA (yellow), whereas the other half was stained for nuclei (blue), cytoskeletons (green) and the endothelial marker CD31 (magenta), scale bar  =  100 µm for all. Higher magnification cLSM image and an orthogonal view (day 7) demonstrate the formation of a closed HUVEC monolayer (magenta) on top of a dense HUVSMC layer (green); scale bar  =  20 µm for both (D).

Aiming to recapitulate the native vascular structure, it was investigated whether a bi‐layered co‐culture of smooth muscle cells and endothelial cells can be generated on the AlgMC/PCL composite scaffolds functionalized with FFP. Human umbilical vein smooth muscle cells (HUVSMC) were seeded onto a NHDF layer previously cultured on the scaffolds and treated with EtOH. The HUVSMC were cultivated in their specific growth medium for 21 days. Live cell staining was used to visualize the HUVSMC on scaffolds at day 1, 7, 14, and 21 of cultivation to monitor their development over time (Figure S8, Supporting Information). Surprisingly, the initial attachment of HUVSMC on scaffolds with EtOH‐treated NHDF layer was poor, with few round cells dispersed at day 1. During cultivation, the cell number increased but did not reach a confluent cell layer. Many, though not all, HUVSMC acquired an elongated shape, and some aligned along the PCL fibers (Figure S8, Supporting Information). Nevertheless, HUVEC were seeded on top of the HUVSMC culture on day 21, and cultivation was continued for up to 7 days in endothelial cell growth medium (**Figure** **8**A). After 1 and 7 days, samples were fixed, cut in half, and HUVMSC as well as HUVEC were stained for their specific markers in the two parts of the sample and imaged at the margin of division. At day 1, HUVEC attached on the scaffold in a dense widespread layer marked by the abundant membranous CD31 expression, while the HUVSMC expressing α‐smooth muscle actin (αSMA) in the cell body were visible as rounded cells (Figure 8B). After 7 days of co‐culture, the HUVEC grew in a fully developed confluent monolayer (Figure 8C). Remarkably, large, elongated cells were visible in the green channel visualizing the actin cytoskeletons – these structures were positive for αSMA staining and could be identified as HUVSMC (Figure 8C). The orthogonal view of a z‐stack obtained by confocal laser scanning microscopy (cLSM) confirmed that the HUVEC were positioned in a monolayer on top of the HUVSMC (Figure 8D). Interestingly, while the HUVSMC in monoculture showed limited adhesion and expansion over time on scaffolds with EtOH‐treated NHDF (Figure S8, Supporting Information), a dense layer could be observed after 7 days of co‐culture with HUVEC (Figure 8D), indicating a supportive effect of the endothelial cells. Further experiments revealed that the EtOH‐treated NHDF layer may preclude adhesion and development of HUVSMC in comparison to FFP‐functionalized AlgMC/PCL composite scaffolds without NHDF as the HUVSMC also tend to attach to the PCL fibers (Figure S9, Supporting Information). The bi‐layered co‐cultures of HUVSMC and HUVEC could also be achieved by seeding the HUVSMC directly on the surface of FFP‐functionalized AlgMC/PCL composite scaffolds, circumventing the use of NHDF feeder layers; the number of HUVEC seemed to be a defining factor of an effective bi‐layer establishment. (Figure S10, Supporting Information).

To summarize the main points, bi‐layered co‐cultures of smooth muscles cells and endothelial cells could be obtained by sequential seeding of HUVSMC and HUVEC on FFP‐functionalized AlgMC/PCL composite scaffolds w/ and w/o an EtOH‐treated NHDF layer. The presence of the NHDF layer impeded adhesion and development of HUVSMC, while the co‐culture with HUVEC compensated for this effect.

## 3 Discussion

Small diameter vessels are hierarchical multilayered constructs: The *tunica intima* on the luminal side consists of an endothelial cell monolayer, attached to a basement membrane that is primarily composed of type IV collagen and laminin, and a fine network of connective tissue with elastic fibers. The *tunica media* as middle part consists of vSMC and type I collagenous and elastic fibers, and provides structural support to the vessel walls. The *tunica adventitia* is the outer covering, predominantly composed of fibroblasts which deposit type I collagen and other extracellular matrix (ECM) components.^[^ ^29^ ^]^ In order to recreate such a hierarchical structure, efforts are made to combine different biomaterials as well as fabrication techniques to take advantage of their specific properties. Following this strategy, in the present work, we have successfully incorporated MEW PCL fiber meshes into 3D printed hydrogel structures to reinforce the mechanical stability, enabling suturing and perfusion of the 4D printed grafts.

The strategy to physically support hydrogels with MEW‐fabricated synthetic polymer fibers has been described as a promising advancement in tissue engineering, enabling to strengthen hydrogel materials that could mimic the natural microenvironment but are otherwise frail.^[^ ^25^, ^30^ ^]^ The challenge in the present study was to reinforce the hydrogels with MEW PCL meshes while maintaining the shape‐morphing effect of the 3D printed structures. The incorporation of the PCL fibers affected the shape morphing significantly, with higher PCL content having a more pronounced impact on the assembly of the structures. Additionally, the geometry of the PCL fiber mesh, which results from the orientation of the fiber placement, was demonstrated to be a crucial factor. Reproducible folding into the desired small diameter tubular structures was only observed when a specific rhombohedral pattern was incorporated; other geometries re‐directed or impeded the acquisition of the final shape (Figure S1, Supporting Information). The underlying mechanism of the shape morphing is a combination of crosslinking and anisotropic swelling of the AlgMC hydrogel structure driven by the surface topography.^[^ ^12^, ^30^, ^31^ ^]^ The densely printed base layer and the pattern of the stripes result in differential swelling of the two scaffold surfaces, generating a bending force.^[^ ^30^ ^]^ Hence, the hydrogel surface with the lower (or slower) swelling behavior becomes the inner lumen of the tubular structure. By incorporating PCL meshes, two key properties of these surfaces were modified: the stiffness of the surfaces and the speed of their swelling behavior, possibly due to the hydrophobic PCL, which may have reduced the water contact angle of the material composition. This observation is supported by a study in which a similar 4D printing approach was investigated: Constante et al. utilized 4D printing by combining 3D extrusion printing of a photo‐crosslinkable methacrylated alginate hydrogel and MEW of PCL fibers, focusing on engineering muscle tissue bundles. A composite was fabricated which consisted of a uniform UV‐treated air‐dried hydrogel and parallel PCL strands. In contrast to our work, the scrolling of this bi‐layer into a tubular structure was driven by inhomogeneous swelling of the hydrogel network with a gradual crosslinking degree, resulting from different exposure to light across the depth of the scaffold.^[^ ^32^ ^]^ As in our study, an influence of the thickness of the PCL layer on the tube formation was observed, with a higher thickness resulting in a higher tube diameter. Building on these findings, future studies will systematically investigate parameters such as PCL mesh density and thickness, as well as the concentration of alginate combined with MC, across both scaffold surfaces. MC plays a key role in ink printability and shape morphing, and its gradual leaching during cell culture may further influence structural integrity and biological responses. This will help deepen our understanding of the complex interplay between these factors and their combined impact on the scaffold's mechanical behavior and functional performance.

Mechanically, the addition of two thin MEW PCL (each max. 30 µm) meshes fulfilled the fundamental requirements for artificial vascular grafts: sutureability and pressure resistance during perfusion. A number of MEW‐printed mesh configurations were tested in this study to choose the final design of the shape‐morphing structures. Nonetheless, further refinement of the PCL fiber diameter and mesh density is needed to enhance mechanical robustness^[^ ^33^, ^34^ ^]^ without compromising the unique shape‐morphing properties. While our proof‐of‐principle suturing and perfusion tests demonstrate initial feasibility, additional mechanical characterization, especially according to clinically relevant standards such as ISO 7198, including luminal tensile strength and burst pressure testing, is essential to ensure suitability for clinical application.^[^ ^27^, ^28^ ^]^

Comparing the mechanical properties of vascular graft substitutes more generally remains challenging due to significant variability in testing methods, including differences in specimen geometry, deformation rates, and analytical approaches. Mechanical parameters such as Young's modulus, compliance, and burst pressure are often derived from fundamentally different experimental setups (e.g., tensile vs hydrostatic burst testing), complicating direct and physiologically relevant comparisons.^[^ ^27^ ^]^ Although similar or even superior mechanical properties have been achieved previously in tissue‐engineered small‐diameter vascular grafts,^[^ ^35^, ^36^, ^37^, ^38^, ^39^ ^]^ none of these promising approaches have, to our knowledge, successfully advanced into clinical translation. One issue might be the often‐used cytotoxic solvents and/or photo‐initiators within the biomaterials, which are necessary to obtain the required tubular shape but aggravate a clinical approval. In contrast, the application of MEW omitted the cytotoxic solvents used in solution electrospinning. Moreover, MEW scaffolds modulated cell adhesion by providing topographical cues that enable directed cell growth, as demonstrated in previous studies.^[^ ^32^, ^36^, ^40^, ^41^ ^]^ The successful merging of the PCL mesh with the hydrogel prevented delamination, though in some areas the PCL fibers were partially covered by the hydrogel (Figure 2Cvi + 5Aii), aggravating a directed cell growth. Nevertheless, a supportive effect of the PCL fibers on cell attachment was observed, albeit only for the fibroblasts (Figure S2, Supporting Information) and not for the endothelial cells (Figure 5B).

Enhancing cell spreading and growth was a critical goal in this study as the employed biomaterials lack cell adhesion cues, especially the AlgMC, which does not support cell attachment. The blending of AlgMC with fibrinogen hindered the shape‐morphing process, but together with the PCL fiber mesh synergistically contributed to a strong enhancement in adhesion and proliferation of fibroblasts and enabled the formation of a dense cell layer on the surface of the composite scaffolds (Figure S2, Supporting Information). More promising was the protein functionalization of the already manufactured grafts by rehydrating the scaffolds in protein‐rich solutions. This procedure avoids the negative effect of the fibrinogen addition to AlgMC on its shape‐morphing ability and can enhance cell attachment to the PCL fibers, which were primarily coated in this procedure. The functionalization with the human blood derivatives FFP and PL proved to be favorable for adhesion and proliferation of fibroblasts on the surface of the AlgMC/PCL composite scaffolds, in contrast to animal‐derived serum functionalization (FCS: 10% in cell culture medium or 100%) which showed no supportive effect (Figures S3 and S4, Supporting Information). Serum, the liquid phase acquired after blood sample coagulation, does not contain fibrinogen or platelets, whereas plasma is generated by centrifuging blood with an added anticoagulant, which preserves the levels of platelets and fibrinogen.^[^ ^42^, ^43^ ^]^ Rapid spinning of plasma results in platelet‐poor plasma (PPP) or, if frozen, FFP^[^ ^43^ ^]^ that is rich in proteins such as fibrinogen, fibronectin, and albumin as major components and still contains growth factors and cytokines.^[^ ^26^, ^44^ ^]^ FFP has been already used to functionalize AlgMC for bioprinting approaches; an improvement in viability, adhesion and spreading, proliferation, and differentiation has been shown for numerous cell types including fibroblasts in bioprinted constructs.^[^ ^26^, ^45^ ^]^ With slow centrifugation of plasma, platelet‐rich plasma (PRP) is obtained; repeated freeze‐thaw cycles result in platelet destruction and release of their content, making PL an abundant source of growth factors and cytokines.^[^ ^46^ ^]^ Since the improved fibroblast adhesion and proliferation on FFP‐ and PL‐rehydrated scaffolds was comparable, the observed effect could be attributed to the coating of AlgMC/PCL scaffolds with fibrinogen and fibronectin, providing cell adhesion sites. Interestingly, collagen appeared to coat the PCL fibers exclusively, causing the fibroblasts to attach and proliferate selectively on them (Figure S3, Supporting Information). Constante et al. coated the 4D printed composite structures of methacrylated alginate and PCL fibers with a mixture of fibronectin, collagen, and albumin prior to seeding the C2C12 myoblasts and also observed adhesion and proliferation of the cells predominantly on the PCL fibers, resulting in a high degree of orientation of the cells by the PCL fiber‐patterned surface.^[^ ^32^ ^]^

In contrast to the positive effect of the protein coating on the colonization of AlgMC/PCL scaffolds by fibroblasts, it did not stimulate HUVEC to form a dense cell layer. Other studies reported that HUVEC adhere to cell culture surfaces coated with fibrin/fibrinogen^[^ ^47^ ^]^ or fibronectin.^[^ ^48^ ^]^ The FFP‐derived protein coating of AlgMC may resemble the blood clot formed in the first step of wound healing, stimulating endothelial cell migration and capillary‐like tube formation – processes that occur during angiogenesis in the wound area during healing – rather than the formation of a closed endothelial cell monolayer covering the inner lumen of blood vessels. In line with this theory, it has been reported that the typical cobblestone‐like monolayer architecture of cultured vascular endothelium is rapidly disorganized after contact with a fibrin clot to initiate cell migration.^[^ ^49^ ^]^ In addition, fibrin matrices are well known to support the pre‐vascular tube formation of endothelial cells^[^ ^50^, ^51^ ^]^ and indeed, formation of tube‐like structures was observed after seeding and culturing HUVEC on FFP‐coated AlgMC/PCL composite scaffolds (Figure 6A).

During the wound healing process, fibroblasts are stimulated by growth factors together with fibrin and fibronectin to migrate into the wound bed, where they proliferate and establish an ECM which supports the restoration of the vascular system and consists mainly of type I collagen, but also includes the basement membrane components type IV collagen and laminin.^[^ ^50^, ^52^, ^53^, ^54^, ^55^ ^]^ Hence, it can be assumed that the fibroblasts growing on the FFP‐coated AlgMC/PCL composite scaffolds deposited a collagen‐based ECM with adhesive proteins that more closely resemble the natural environment of endothelial cells in blood vessels. Subsequently, the seeded HUVEC on such a fibroblast feeder layer formed a continuous layer displaying the typical cobblestone morphology (Figure 7; Figure S6, Supporting Information). This endothelial layer formation was observed on both live and EtOH‐treated NHDF layers, suggesting that the deposited ECM is the supportive factor rather than biochemical signaling molecules secreted by live fibroblasts. Interestingly, in other endothelial cell/fibroblast co‐culture systems, the attachment of endothelial cells to the matrix‐rich surface of fibroblasts was followed by the formation of tube‐like structures.^[^ ^55^ ^]^ In that case, VEGF as key angiogenic factor, was present in the cell culture medium. Thus, the absence of soluble pro‐angiogenic factors in our study – no VEGF was added to the medium, and the EtOH‐treated fibroblast feeder layer did not secrete growth factors – may have promoted the formation of a stable endothelial layer with the typical cobblestone‐like morphology (Figure 7; Figure S7, Supporting Information).

To recapitulate the tri‐layered structure of vessels, smooth muscle cells were pre‐cultured on an EtOH‐treated NHDF feeder layer before seeding the HUVEC. Surprisingly, initial adhesion as well as growth of HUVSMC on the feeder layer were low; later experiments revealed a better colonization of FFP‐rehydrated composite scaffolds by HUVSMC without the fibroblast layer (Figures S8 and S9, Supporting Information). The reason for the reduced HUVSMC adhesion and proliferation on the fibroblast layer is unclear. One could argue that dermal fibroblasts are insufficient in contrast to vascular fibroblasts. On the other hand, Baba et al. used a similar sequential seeding approach of fibroblasts (NHDF), smooth muscle cells (human aortic; hASMC), and endothelial cells (HUVEC) onto glass fiber sheets and generated tri‐layered structures whereby the fibroblasts showed invasive growth with penetration into the material and both the hASMC and HUVEC formed cell layers on top of the previously seeded cell type.^[^ ^56^ ^]^ In that work, the tri‐layered structures were seeded either with dermal fibroblasts or vascular fibroblasts. After a dynamic cultivation, both types of fibroblasts showed similar histological structure and mechanical properties, indicating that vascular fibroblasts can be replaced by dermal fibroblasts, with the advantage that they are easier to acquire from autologous sources and exhibit a superior growth rate and replicative potential in 2D culture.^[^ ^56^ ^]^

Regardless, once the HUVEC were seeded on top of the HUVSMC, a robust bi‐layered cell structure developed during further cultivation, consisting of a confluent endothelial cell monolayer and an underlying dense layer of smooth muscle cells with elongated αSMA‐positive structures (Figure 8). These observations further underline the strong supportive character between both cell types when cultured in vitro.^[^ ^57^, ^58^ ^]^

Furthermore, the synthetic or contractile phenotype of smooth muscle cells could previously be induced if grown together with an expanding or a confluent endothelial cell layer, respectively.^[^ ^59^ ^]^ Proliferating synthetic smooth muscle cells exhibit a cobblestone morphology and low αSMA expression, while contractile smooth muscle cells are elongated and spindle‐shaped with aligned αSMA filaments.^[^ ^60^ ^]^ This may explain the observed gradual phenotypic shift of HUVSMC from rounded αSMA‐positive cells at day 1 to elongated, contractile morphologies by day 7, potentially induced by the formation of a confluent endothelial monolayer (Figure 8). Reciprocally, HUVSMC appeared to support endothelial layer formation even in the absence of a fibroblast feeder layer, suggesting a bidirectional stabilizing interaction (Figure S10, Supporting Information) and opening the option to seed the fibroblasts on the outer side of the tubes to achieve the tri‐layered vessel structure in the future. These findings reflect established observations regarding endothelial‐smooth muscle crosstalk in MEW scaffold‐supported systems.^[^ ^25^, ^36^ ^]^

Taken together, our results contribute to the evaluation of hybrid fabrication strategies for small‐diameter tissue‐engineered vascular grafts. While our combination of 4D printing and MEW enables the integration of dynamic shape morphing with topographical guidance of cell attachment, it remains limited in addressing key requirements such as mechanical compliance and sustained phenotypic maintenance. Compared to solution electrospinning/MEW hybrids,^[^ ^61^ ^],^ volumetrically printed hydrogel constructs,^[^ ^38^ ^]^ or electrospun bi‐layers with tunable stiffness,^[^ ^62^ ^]^ the presented approach offers a broader conceptual design space but currently falls short in terms of structural robustness and biological integration.

Future refinements should focus on enhancing mechanical performance without compromising the morphing functionality. Tailoring MEW fiber diameter, fiber architecture, and surface properties could further improve smooth muscle cell alignment and stabilization, while maintaining the shape‐morphing effect. Moreover, the use of (autologous) mesenchymal stem cells instead of terminally differentiated HUVSMC could offer a renewable source of cells and promote a more efficient mechanosensitive maturation toward contractile phenotypes following the scaffold's topographical cues.

Taken together, these developments could help reposition this 4D printing‐based AlgMC/PCL platform—not necessarily as a direct solution for vascular grafting—but as a promising strategy for soft tissue constructs that rely on dynamic form adaptation, spatially controlled adhesion, and guided cellular architecture.

## 4 Conclusion

This study presents a comprehensive evaluation of a hybrid fabrication strategy combining 4D printing of AlgMC hydrogels with MEW PCL reinforcement for the development of shape‐morphing, small‐diameter tubular constructs. Integrating MEW fiber meshes into the hydrogel matrix significantly improved the mechanical stability of otherwise fragile hydrogel constructs, thereby fulfilling principal requirements such as sutureability and pressure resistance during perfusion. The post‐manufacturing functionalization of the composite scaffolds with blood‐derived protein coatings significantly improved cellular adhesion and viability, particularly for fibroblasts, while allowing to preserve the shape‐morphing ability of the system.

An in‐depth biological characterization was conducted using mono‐ and co‐cultures of fibroblasts, endothelial cells, and smooth muscle cells. Endothelial and smooth muscle cells formed organized bi‐layered structures with phenotype‐specific morphology and evidence of reciprocal stabilization. Notably, the presence of confluent endothelial layers promoted smooth muscle cell elongation and αSMA expression, while smooth muscle cells enhanced endothelial coverage even in the absence of a fibroblast feeder layer. These findings highlight both the potential and the current limitations of the platform, particularly in achieving stable endothelialization. Nonetheless, the system provides a versatile basis for further development of tissue‐engineered constructs that rely on dynamic architecture, spatial control of cell adhesion, and guided cell organization.

## 5 Experimental Section

### Extrusion‐Based 3D Printing of the Hydrogels

The alginate‐methylcellulose ink (AlgMC) was prepared by dissolving 3% (w/v) of alginic acid sodium salt (Sigma Aldrich, Taufkirchen, Germany) in double‐deionized water at room temperature using a magnetic stirrer. Once the alginate was fully dissolved, the solution was removed from the stirrer, and 9% (w/v) methylcellulose powder (approximate MW = 88 kDa, Sigma Aldrich) was added. The alginate‐methylcellulose ink supplemented with fibrinogen (AlgMC + F) was prepared by dissolving the desired amount (2.5 mg ml^−1^, 5.0 mg ml^−1^, 10.0 mg ml^−1^, 20.0 mg ml^−1^, 40.0 mg ml^−1^) of freeze‐dried fibrinogen powder (fibrinogen from bovine plasma, Sigma Aldrich) in double deionized water at room temperature using a magnetic stirrer. Then, 3% (w/v) of alginic acid sodium salt was added and stirred until fully dissolved. The solution was then removed from the stirrer, and 9% (w/v) methylcellulose (MC) powder was added. For both the AlgMC and the AlgMC + F inks, mixing was done manually using a spatula until homogeneous. To let the methylcellulose hydrate, the hydrogel precursor, was stored overnight at room temperature. For the cell culture experiments, ink preparation was carried out under sterile conditions, using autoclaved alginate and methylcellulose powders and sterile‐filtered fibrinogen solution.

The inks were 3D printed using the BioScaffolder 3.1 (GeSiM mbH, Radeberg, Germany) through conical nozzles with an outlet diameter of 410 µm or 200 µm. Printing pressures, especially dependent on the nozzle size, ranged from 160–450 kPa, the printing speed ranged from 7–10 mm s^−1^, and the chosen strand width, layer height, and z‐offset were the same dimensions as the inner needle diameter (410 or 200 µm). The printed structure consisted of three layers, including one solid base layer and two identical layers of lines, created from single strands with an interspace, plotted on top of each other (Figure 1A). While the base layer was printed from left to right (± x‐direction), the experiments showed that it was necessary to invert the direction of the lines (top to bottom, ± y‐direction) to achieve a shape‐morphingeffect. The interspace between the lines was adjusted as stated in the respective chapter.

### Melt Electrowriting of the PCL Meshes

Medical grade polycaprolactone (PCL, Purasorb PC 12 Corbion, Amsterdam, Netherlands) was filled into a metal cartridge, heated to 73 °C and printed with the BioScaffolder 3.1 (GeSiM) equipped with a MEW‐module. Fibers with a diameter of 7 ± 1.5 µm were obtained with a printing speed of 1440 mm/min, a voltage of 6.15 kV, a pressure of 7 kPa and a nozzle to printing bed distance of 2.6 mm. The inner nozzle diameter was 250 µm.

### Combined Melt Electrowriting and Extrusion Printing of Composite Structures

A multistep process was established to fabricate the composite structures (Figure 2A): First, a PCL mesh was printed, followed by the hydrogel base layer, which was allowed to dry for 30 min before a second PCL mesh (identical to the first) was printed on top. Lastly, two layers with hydrogel stripes were printed.

### Shape‐Morphing and Crosslinking

After air‐drying overnight, the printed hydrogel or composite structures were immersed into 0.1 m calcium chloride solution (Sigma Aldrich) to initiate shape morphing into tubular structures by hydrogel swelling. In case of AlgMC + F, 3 U mL^−1^ thrombin (Baxter, Heidelberg, Germany) was added to the 0.1 m calcium chloride solution. Time of immersion depended on the shape‐morphing behavior of the structure as stated in the respective chapter. Following freeze‐drying, an additional crosslinking step was carried out in 1 m calcium chloride solution for up to 2 h to ensure complete crosslinking and maximum structural stabilization of the constructs.

### Freeze Drying and Storage

Structures were removed from the crosslinking solution, carefully patted dry using a filter paper, and then frozen at −20 °C for least 24 h. After freeze‐drying overnight, the tubular structures were stored at room temperature.

### Mechanical analysis

Tensile tests were performed with a uniaxial testing machine (Z010, ZwickRoell, Ulm, Germany) equipped with a 100 N load cell. Tubular scaffolds (length: 30 mm) were fixed with customized adapters. The adapters were designed with Solidworks CAD software and followed the screw grip methods of the manufacturer of the testing machine for forces up to 20 N. The red adapter was printed with polylactic acid (Prusament, Prusa Research, Prague, Czech Republic) by Prusa MK3S+ (Prusa Research). Tests were run until breakage with a velocity of 1 mm min^−1^. The young's modulus was assessed by measuring the linear slope of the obtained stress/strain curves, similar to previous studies.^[^ ^63^ ^]^ The ultimate tensile strength reflects the maximum force measured after the linear slope.

### Sewing Test

A sewing kit of 6–0 Prolene Polypropylene thread and needle (Ethicon, Bridgewater, New Jersey, USA) was used. Directly after crosslinking, the tubes were transferred into a plastic petri dish. They were punctuated with the needle, which was then pulled through the wall of the composite tubes. A knot was then tied to test whether the thread could be fixed in the material or would cut through it when tension was applied. If the knot could be made, the tube was divided into two halves and sewn together by making several individual knots. The sewn tubes were transferred to the perfusion test.

### Perfusion test

Water containing food coloring was filled into a pipe system connected to a pulsatile pump (ISM945, ISMATEC, Cole‐Parmer, Wertheim, Germany). The ends of the pipe system were then inserted into the ends of hydrated tube samples, taken directly after crosslinking or after sewing (Figure 4); the pipes were inserted either directly or by the addition of conical printing nozzles (Nordson EFD, Oberhaching, Germany), which were used as adapter to match the tube diameter. To test the sample for leak‐tightness, the pump was run at full speed, which, with the chosen tube system, equals ≈1.5 mL min^−1^.

### Fabrication of Flat Model Scaffolds for Cell Culture Experiments

Composite scaffolds were prepared from PCL fiber meshes and either plain AlgMC or AlgMC + F hydrogel scaffolds (fibrinogen concentration: 5 mg mL^−1^). To test the cell interactions with the (modified) materials, flat model composite scaffolds were fabricated in a simplified two‐step approach. First, PCL meshes with a fiber diameter of ≈7 µm, a 60° layer‐to‐layer orientation, and an adjustable fiber interspace were fabricated. Their stability for handling was increased with a fused deposition modeled solid frame (13 × 13 mm) from PCL melted at 95 °C using a BioScaffolder 3.1. equipped with a MEW‐module (GeSiM). After fabrication, the meshes were removed from the printing bed, hydrophilised in 5 m NaOH (131 687.1211, AppliChem) for 1 h, washed with distilled water, and disinfected by immersion into 70% ethanol. Second, sterile hydrogel precursor squares of 13 × 13 mm were printed in a laminar flow hood using the BioScaffolder 3.1 (GeSiM) into sterile well plates. Subsequently, the PCL meshes were manually placed on top, and the composite samples were left to dry at room temperature overnight. For crosslinking, the AlgMC + F‐based samples were immersed into 0.1 m calcium chloride and 3 U mL^−1^ thrombin solution for about 30 mins, and the AlgMC‐based samples in 70 mm strontium chloride (Roth, Karlsruhe, Germany) for increased stability instead. Afterward, the samples were freeze‐dried overnight at −40 °C at low pressure using an Alpha 1–2 lyophiliser (Christ, Osterode, Germany) and stored at room temperature in sterile conditions.

### Protein Functionalization

The freeze‐dried scaffolds were oriented with the PCL mesh facing upward and were rehydrated in protein‐rich solutions and air‐dried overnight, immediately before seeding with cells. For this, the samples were treated with cell culture medium consisting of Dulbecco's Modified Eagle Medium (DMEM; Gibco, Thermo Fisher Scientific, USA) with 10% fetal calf serum (FCS; Corning Inc., NY, USA) as well as 100 U mL^−1^ penicillin and 100 µg mL^−1^ (P/S; Gibco), 100% FCS, human fresh frozen plasma (FFP), human platelet lysate (PL), collagen solution from human fibroblasts (150 µg mL^−1^, Sigma‐Aldrich), or gelatin‐based coating solution (0.1%, CellBiologics, Chicago, IL, USA). The PL was prepared from human platelet‐rich plasma (PRP) as described elsewhere, by lysing platelets in four freeze‐thaw cycles, removing cell debris by centrifugation at 17,500 g for 20 min and filtering through low protein binding filters^[^ ^64^ ^]^ The FFP and PRP were acquired from the German Red Cross (BSD Ost, Dresden, Germany) and used as a pool of five and twenty donors, respectively, to reduce the variation between individuals.

### Cell Culture Experiments With Normal Human Dermal fibroblasts

NHDF, purchased from PromoCell GmbH (Heidelberg, Germany), were cultured in DMEM with 10% FCS and P/S under standard culture conditions (37 °C, 5% CO_2_, 95% humidity). The scaffolds were seeded immediately after crosslinking and rinsing with HBSS or after protein functionalization by adding 100 µl cell suspension containing 1‐1.5 × 10^5^ cells. After 1 h of initial attachment, cell culture medium was added. During cultivation, the medium was refreshed twice per week.

### Cell Culture Experiments With Human Umbilical Vein Endothelial Cells

HUVEC, purchased from PromoCell GmbH were cultured in endothelial cell growth medium (PromoCell GmbH) under standard culture conditions. The scaffolds were seeded immediately after crosslinking and rinsing with HBSS or after protein functionalization by adding 100 µl cell suspension containing 1–1.5 × 10^5^ cells. After 2 h of initial attachment, endothelial cell growth medium was added. For establishing an NHDF feeder layer, the NHDF (1 × 10^5^/scaffold) were seeded and cultivated in DMEM with 10% FCS and P/S until near confluence (for 7–9 days). The HUVEC (2.3 × 10^5^/scaffold) were seeded either directly onto the live NHDF layer or after treatment of the NHDF with 96% ethanol for 5 min followed by aspiration and air‐drying overnight. During cultivation, the medium was refreshed twice per week.

### Co‐Culture Experiments With Human Umbilical Vein Smooth Muscle Cells and Human Umbilical Vein Endothelial Cells

HUVSMC, purchased from Innoprot (Derio, Bizkaia, Spain), were cultured in smooth muscle cell growth medium (Innoprot) under standard culture conditions. For HUVSMC monoculture, the cells (1.5 × 10^5^/scaffold) were seeded on scaffolds with an EtOH‐treated NHDF layer as described before and cultivated in smooth muscle cell growth medium. For co‐culture experiments, HUVSMC (1.5 × 10^5^/scaffold) were seeded first and, after 21 days of cultivation in smooth muscle cell growth medium, HUVEC (1.2–2.3 × 10^5^/scaffold) were seeded on top; further cultivation was carried out in endothelial cell growth medium. During cultivation, the medium was refreshed twice per week.

### Analysis of Viability

The cell viability was evaluated by simultaneous staining of the cell‐seeded scaffolds with calcein AM and ethidium homodimer‐1 (Invitrogen). The Live/Dead staining was performed by subjecting the samples to culture medium containing the dyes at 2.4 µm concentration. After a 20–30 min incubation at 37 °C, the medium with dyes was replaced with fresh medium, and the stained scaffolds were imaged cell‐seeded side down using a Keyence BZ‐X800 microscope. Image stacks were acquired with 2, 4, and 10X objectives using a quick full focus function and are represented as maximum intensity projections. Whole‐sample overviews, used for quantitative analysis, were formed in Analyser software by stitching images taken with a 2X objective. The cell viability was quantified via ImageJ (version 1.53n, National Institutes of Health, USA) using the Threshold and Analyze Particles functions to determine the area occupied by live and dead cells either within the field of imaging using a 10X objective or in the whole area of the scaffold within the frame.

### Fluorescence Staining

The cell‐seeded scaffolds were fixed with 4% formaldehyde in Hank's balanced salt solution (HBSS) for 30 min at room temperature. The cell membrane was permeabilized with 0.5% Triton X‐100 in HBSS for 5 min. The scaffolds were incubated in a blocking solution with 3% bovine serum albumin (BSA) or goat serum (Gibco) in HBSS for 1 h. The samples containing HUVEC were subjected to a primary monoclonal mouse anti‐human CD31 antibody (1:402, JC70A, Dako) in HBSS with 0.5% goat serum and 0.1% Tween 20 (Sigma‐Aldrich) for 1 h. For HUVSMC‐seeded scaffolds, a primary mouse anti‐alpha‐smooth muscle actin (αSMA) antibody (1:400, A2547, Sigma‐Aldrich) was used for a 3 h incubation. In both cases, a secondary Alexa Fluor 594 goat anti‐mouse IgG antibody (1:500, A11005, Life Technologies) was used for staining in combination with Phalloidin iFluor 488 (1:1000, ab176753, Abcam) and DAPI (1:2000) in HBSS with 0.5% BSA or goat serum for 1 h. For staining of only the cell nuclei and cytoskeletons, samples were only incubated in HBSS with 0.5% BSA containing Phalloidin iFluor and DAPI for 1 h. Image stacks were acquired on a Keyence BZ‐X800 microscope or an inverted Leica SP5 confocal laser scanning microscope using 10X or 20X objectives with a z‐step of 4 µm and using a 63X immersion objective with 1 µm steps. Solely for the investigations presented in Figure 8, a 8 (Leica microscope systems) was stellarised. The images were processed and are presented as maximum intensity projections using ImageJ.

### Statistical Analysis

Graphs were prepared, and statistical analysis was carried out using GraphPad Prism (version 10.0.0 for Windows, GraphPad Software, Boston, Massachusetts, USA). Data in the text and graphs are given as mean ± standard deviation (SD) of at least three values or are stated as exact values in the text, and only a mean line is shown in the graphs if the sample size was smaller. The statistical tests and p‐values are indicated in the figure captions, while the significant differences between groups are represented in graphs with asterisks. All tests were carried out with a level of significance set to 0.05.

## Conflict of Interest

The authors declare no conflict of interest

## Supporting information



Supporting Information

Supplemental Video 1

Supplemental Video 2

Supplemental Video 3

## Data Availability

The data that support the findings of this study are available from the corresponding author upon reasonable request.
