# Supplementary material for: Uniting 4D Printing and Melt Electrowriting for the Enhancement of Regenerative Small Diameter Vascular Grafts
Source: Adv Healthc Mater. 2025 Aug 7;14(30):e02380. doi: 10.1002/adhm.202502380 (PMC12645082; doi:10.1002/adhm.202502380)
Supplement: Supplementary file 1 — Supporting Information [file ADHM-14-0-s004.docx]

Supporting Information

Uniting 4D Printing and melt electrowriting for the enhancement of vascular grafts

Max von Witzleben^#^, Akvile Gasiunaite^#^, Marlene Ihle, Ashwini Rahul Akkineni, Kathleen Schütz, Tilman Ahlfeld, Michael Gelinsky, Anja Lode* and Sarah Duin

Technische Universität Dresden, University Hospital *Carl Gustav Carus* and Faculty of Medicine, Center for Translational Bone, Joint and Soft Tissue Research, Fetscherstraße 74, 01307, Dresden

*E-mail: [Max.von_Witzleben@tu-dresden.de](mailto:Max.von_Witzleben@tu-dresden.de)

^#^shared first authorship

***1.*** ***Effect of PCL MEW mesh properties on shape morphing of printed AlgMC hydrogels***

The geometry of the PCL meshes fabricated by MEW had a strong influence on the shape-morphing effect. The diameter of the fibers was kept constant (7 ± 1.5 µm) while the fiber spacing (100 µm, 200 µm), the number of layers (2, 4, 6), the layer-to-layer orientation (20°, 60°, 90°), and fiber direction were varied. Figure S1 provides on overview of the tested variants with the obtained results – missing images indicate that there was no shape morphing observed. The variant with PCL meshes consisting of four layers, with a 60° angle, and a fiber spacing of 100 µm (Figure S1-8b) achieved the desired structure, was reproducible and therefore was selected for further analysis.


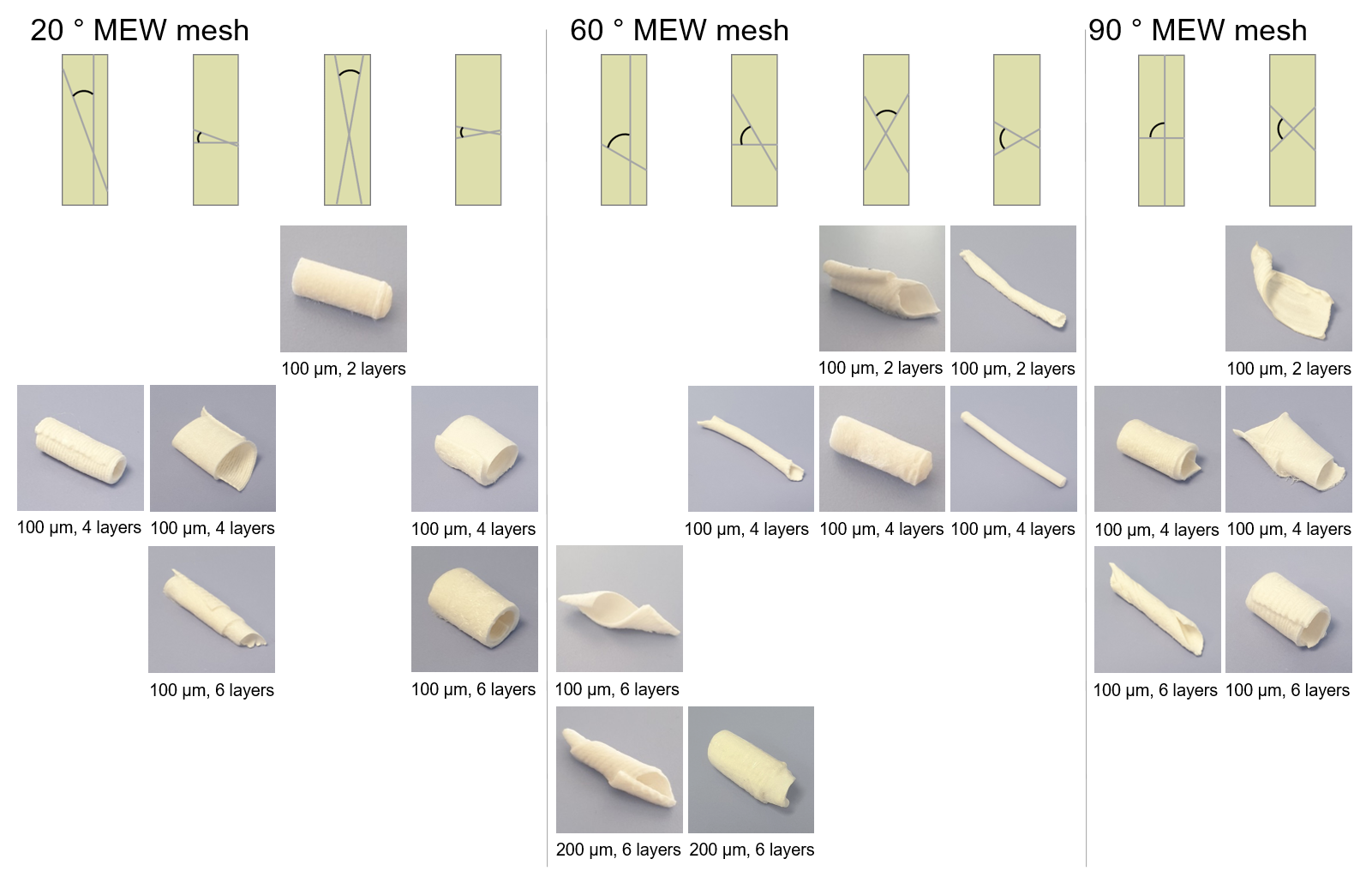


**Figure S1.** Documentation of the shape-morphing behavior of AlgMC hydrogel-PCL mesh composite structures in dependence of the PCL mesh geometry. Schematic on top depicts the flat structure (12.5 mm × 35 mm) in its air-dried state before shape-morphing with two exemplary PCL fibers, showing their orientation and direction. Empty fields indicate no shape-morphing of these variants. The variant shown in 8b (green frame) was selected for further analysis.

***2. Improvement of cell colonization by integration of PCL MEW meshes***

The integration of fibrinogen at a concentration of 5 mg ml^-1^ into AlgMC, which was converted to fibrin during crosslinking in the presence of thrombin, enhanced the adhesion and spreading of NHDF on the surface of AlgMC+F samples in comparison to those consisting of AlgMC without fibrin. However, it was not sufficient to achieve a uniform and complete coverage of the scaffold surface. The strongest enhancement of cell attachment and spreading was observed on the surface of composite scaffolds consisting of AlgMC+F and PCL mesh: NHDF formed a uniform and nearly closed cell layer during 7 days of cultivation (**Figure S2**). The lower magnification image of AlgMC+F with PCL mesh (identical to Figure 5B, Day 7) was intentionally reused in Figure S2 to enhance visual comparability.


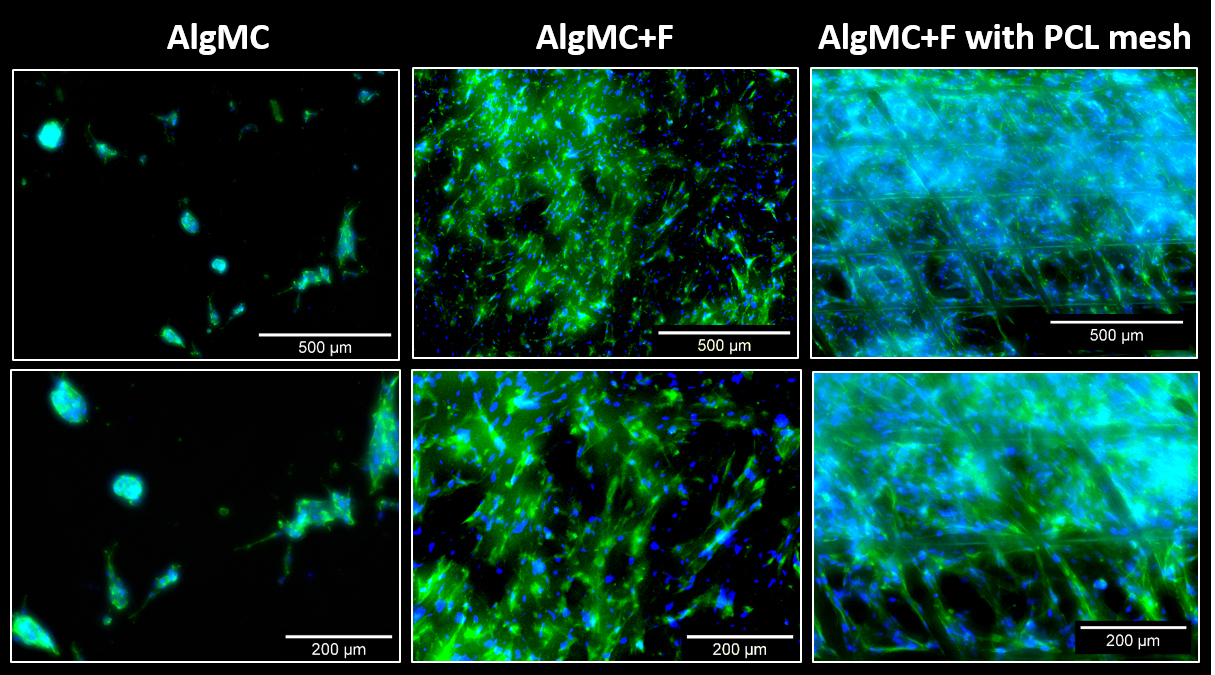


**Figure S2.** NHDF were seeded on flat scaffolds consisting of AlgMC hydrogel, AlgMC hydrogel modified with 5 mg ml^-1^ fibrinogen (AlgMC+F) and AlgMC+F hydrogel combined with PCL mesh. After 7 days of cultivation, cell colonization was assessed by fluorescence-microscopy after staining of the nuclei with DAPI (blue) and the cytoskeletons with Phalloidin 488 (green). Bottom row – magnification of the areas in the top row.

***3. Protein functionalization after fabrication for optimized cell colonization***

Five different compositions were compared regarding their ability to support attachment of NHDF to the composite scaffolds: cell culture medium containing 10 % fetal calf serum (FCS), 100 % FCS, 100 % human fresh frozen plasma (FFP), human collagen type I (150 µg/mL), and bovine gelatin (0.1%). The evaluation of cell adhesion and viability one day after seeding (1x10^5^ cells/scaffold) revealed pronounced differences between protein coatings (**Figure S3**A). The two-hour long rehydration in cell culture medium supplemented with 10 % FCS as well as pure FCS resulted in loosely attached cells which stayed in a round shape. In contrast, the use of FFP had a strikingly positive influence on cell adhesion and spreading on the surface of the scaffold with large areas covered confluently. The collagen primarily coated the PCL fibers and encouraged the cells to spread along the fibers while they stayed round in the areas of exposed AlgMC. A similar outcome was observed on the gelatin-coated scaffolds, where the NHDF also preferentially adhered to the PCL mesh. The efficiency of cell colonization was evaluated by quantifying the density of the cell layer which showed a significant difference between the groups (Figure S3B). Within the field of imaging, around 2.0 ± 1.4 % and 2.3 ± 1.1 % of area was occupied by NHDF on the scaffolds rehydrated in medium and FCS. For collagen- and gelatin-coated samples, the percentage reached 6.6 ± 6.0 % and 6.3 ± 1.8 %, respectively, though the increase was not statistically significant. In contrast, the FFP-rehydrated scaffolds displayed a significantly larger coverage with 57.0 ± 20.3 % of the area populated by cells. The viability of NHDF was high in all groups (Figure S3C). The proportion of live cells on the scaffolds rehydrated in medium, FCS and collagen was comparable, with approximately 96% viability each, while with the FFP the percentage was close to 100%. A slightly lower viability of 87.4 ± 4.3 % was observed on the gelatin-coated scaffolds.


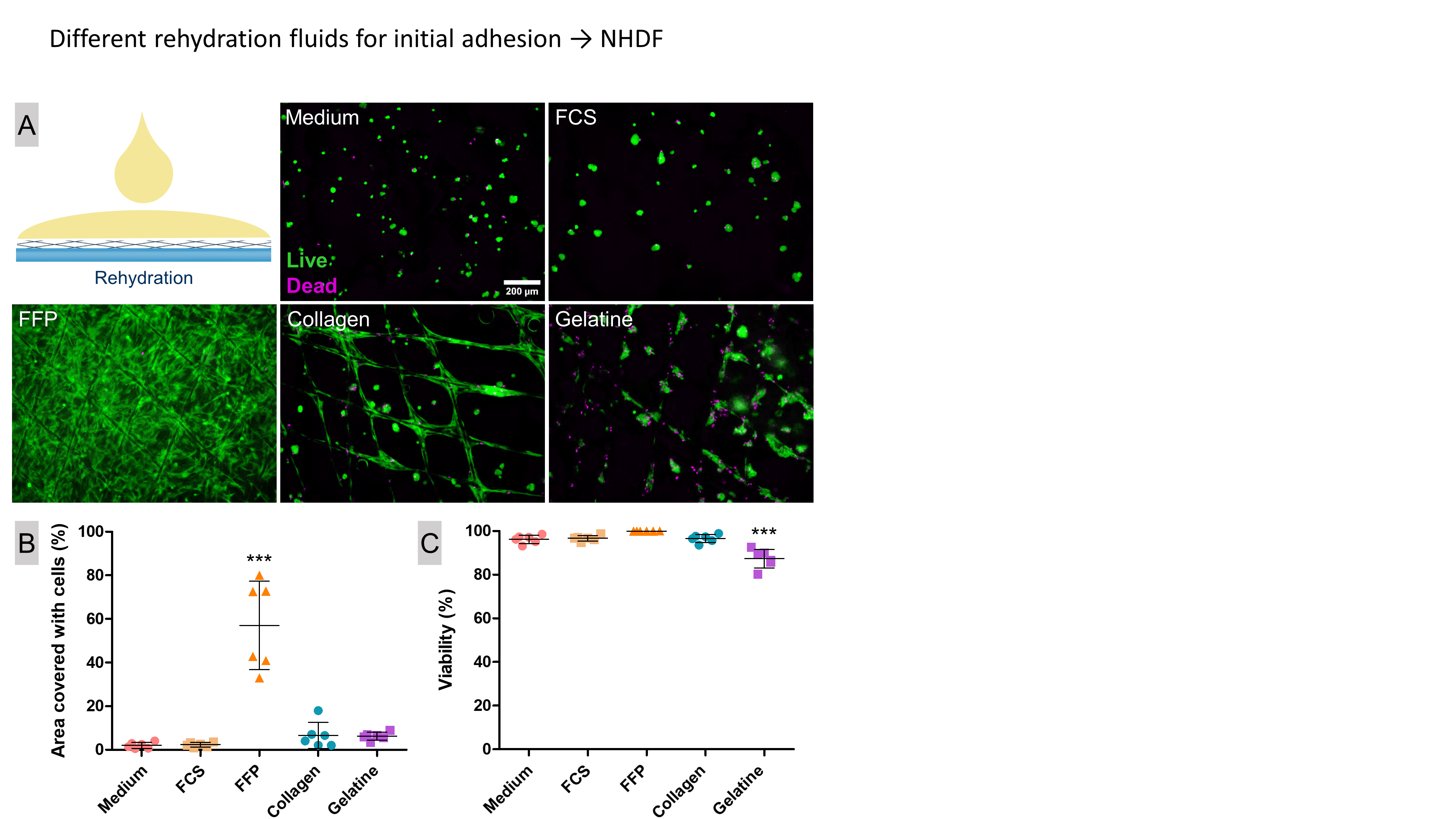


**Figure S3.** Initial adhesion and viability of NHDF seeded on flat composite scaffolds (1.5x10^5^/scaffold) of AlgMC hydrogel and PCL mesh, which were functionalized by rehydration in different protein solutions after freeze-drying. Simultaneous staining of live (green) and dead (magenta) cells was performed after one day of cultivation: (A) exemplary fluorescence-microscopic images, scale bar = 200 µm for all. Percentage of area covered with cells (B) and proportion of viable cells (C) within the field of imaging was determined by analysis of two scaffolds, three sites each (n = 6; mean ± SD). One-way ANOVA, Tukey’s post-hoc test. Significant differences represented as ***p<0.0001.

Following initial adhesion and viability of the NHDF, their ability to proliferate on the scaffolds was assessed. In addition to the collagen and FFP coatings, as the human blood plasma seemed by far the most promising solution for functionalization, platelet lysate (PL) was included into the analysis as another type of human blood derivative rich in growth factors and fibrin (**Figure S4**). On the collagen-coated scaffolds, the cells largely stayed in agglomerates during the 9 days of cultivation (Figure S4A, top row) which resulted in an unchanged area coverage of only about 1% throughout (Figure S4B). In contrast, the NHDFs spread and covered a large area of scaffolds rehydrated in both derivatives of human blood plasma 1 day after seeding (17.6 % to 21.7 % and 10.6 % to 18.0 % for FFP and PL, respectively) (Figure S4A and B). Over time of culture, the coverage of PL-functionalized scaffolds remained constant, but with a very high variability between samples. On FFP-functionalized scaffolds on the other hand, the lower coverage on day 4 and recovery towards day 9 indicates a loss of cells in the first few days, but proliferation following that, and a much lower variation between the samples. On both, FFP- and PL-functionalized scaffolds, NHDF exhibited a very high viability over the cultivation time; the somewhat reduced viability observed on the collagen-coated scaffolds can be explained by the untypical cell clustering (Figure S4C).


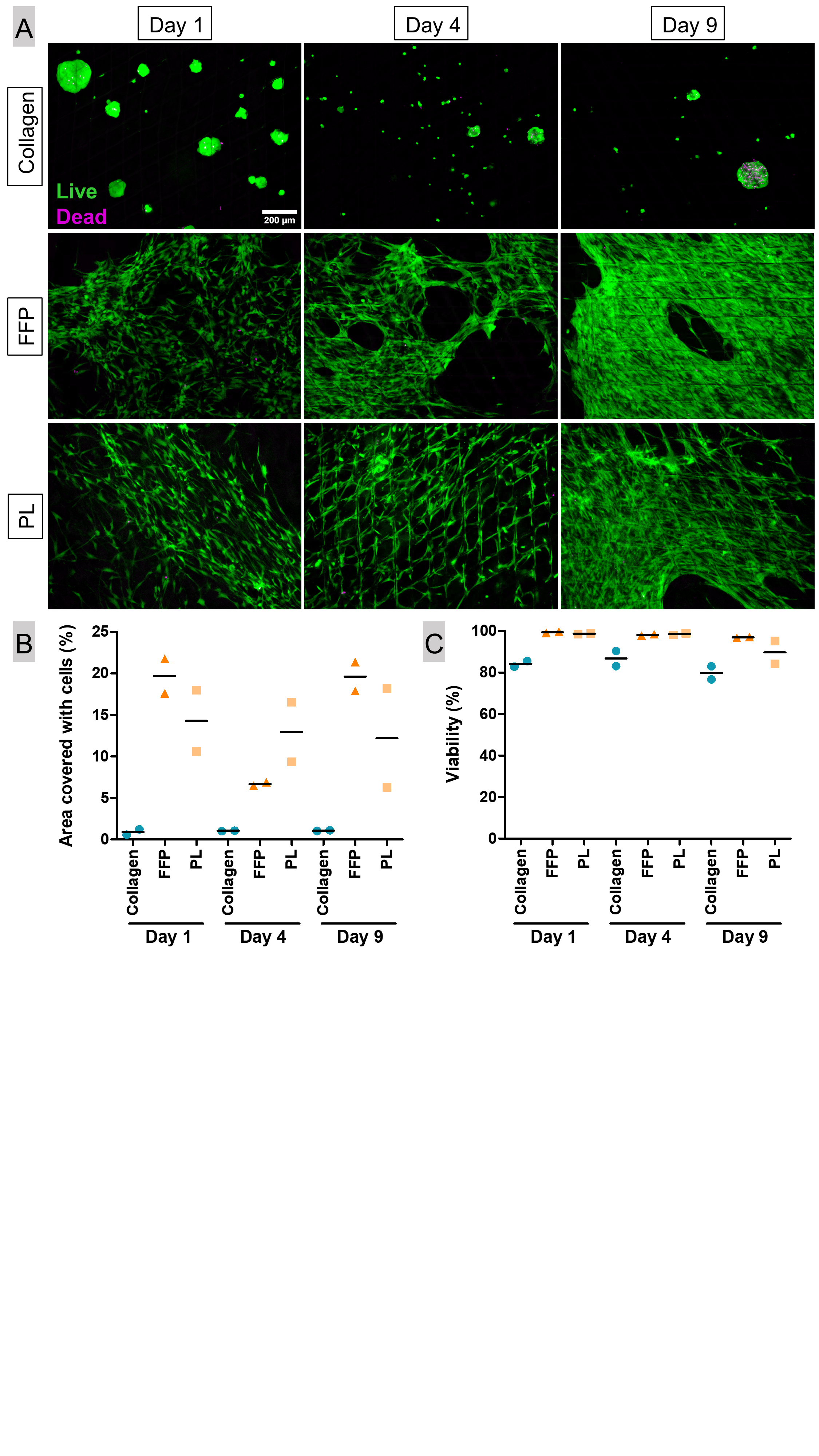


**Figure S4.** Growth and viability of NHDF seeded on flat composite scaffolds (1x10^5^/scaffold) of AlgMC hydrogel and PCL mesh, which were functionalized by rehydration in collagen, FFP or PL solutions after freeze-drying. Simultaneous staining of live (green) and dead (magenta) cells was performed at day 1, 4, and 9 of cultivation: (A) Exemplary fluorescence-microscopic images, scale bar = 200 µm for all. (B) Percentage of the whole scaffold area covered with cells and (C) proportion of viable cells (n = 2; inner area of two scaffolds and their mean).

These coatings were also analyzed for their initial support of HUVEC (**Figure S5**). While NHDF showed promise in forming a monolayer of cells over the scaffolds, results show that the same setup cannot be used to grow HUVEC: They showed a moderate cell adhesion, but many cells stayed round, collected in cell clusters, and did not acquire typical endothelial cobblestone morphology (Figure S5A). Due to the cluster formation, despite an increased viability on day 4, the area coverage was even reduced after 4 days of culture compared to day 1 (Figure S5B and C).


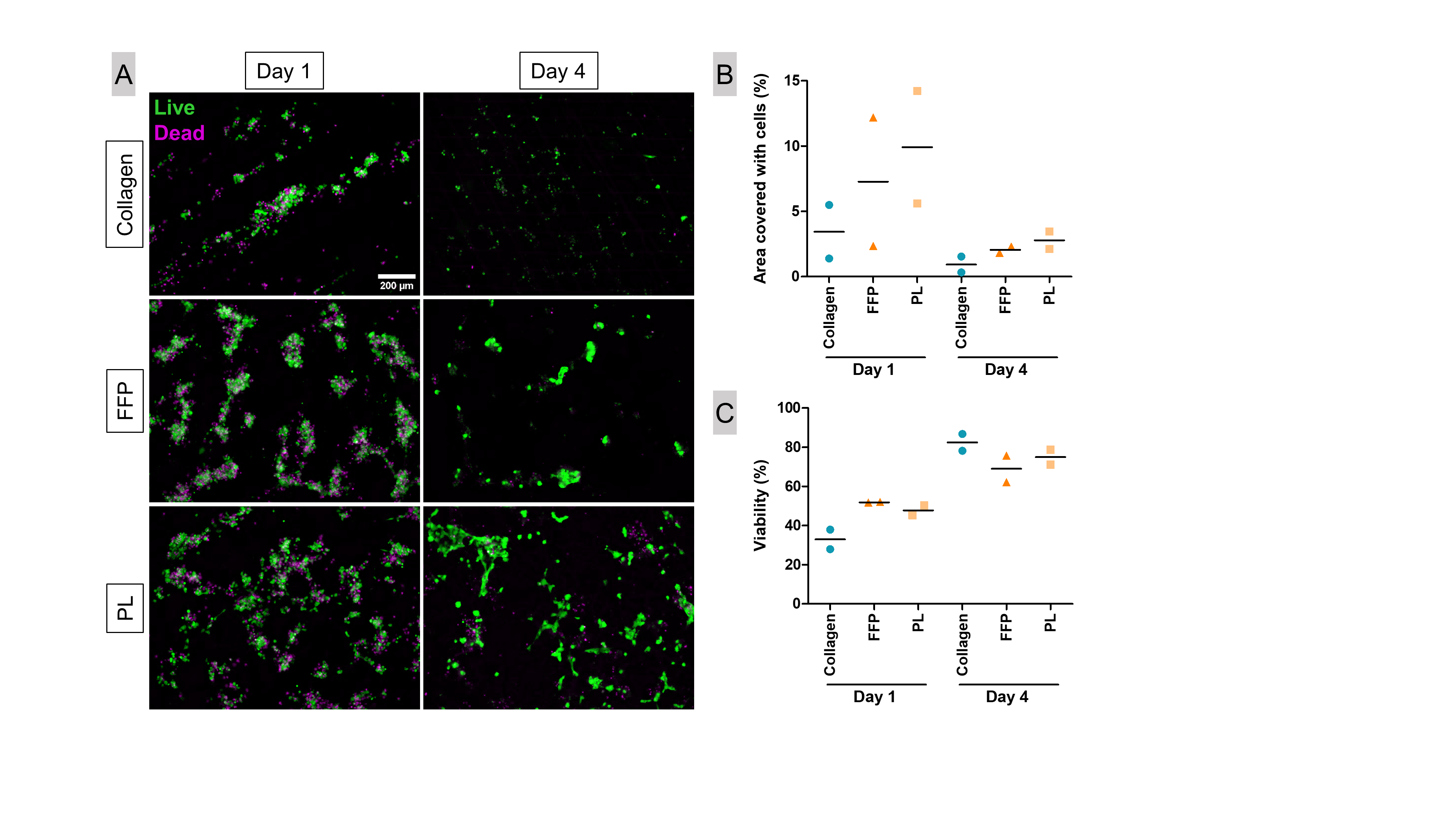


**Figure S5.** Adhesion, growth and viability of HUVEC seeded on flat composite scaffolds (1.5x10^5^/scaffold) of AlgMC hydrogel and PCL mesh, which were functionalized by rehydration in collagen, FFP or PL solutions after freeze-drying. Simultaneous staining of live (green) and dead (magenta) cells was performed at day 1, and 4 of cultivation: (A) Exemplary fluorescence-microscopic images, scale bar = 200 µm for all. (B) Percentage of the whole scaffold area covered with cells and (C) proportion of viable cells (n = 2; inner area of two scaffolds and their mean).

***4. Endothelialization supported by NHDF***

The formation of a confluent HUVEC monolayer was investigated on both live and EtOH-treated NHDF layer. A dense widespread feeder layer of NHDF was established on FFP-functionalized AlgMC/PCL composite scaffolds over a cultivation period of 9-10 days. HUVEC were seeded either directly on top of the live NHDF (**Figure S6**A) or on EtOH-treated and air-dried NHDF layer. The live NHDF supported efficient adhesion and spreading into a tightly packed layer within one day as indicated by CD31 staining. However, during further cultivation they continued to proliferate, and congregated cell masses appeared after 7 and 14 days of cultivation, reducing the expansion of HUVEC layer (Figure S6B). After 14 days, the strongly proliferating NHDF led to the detachment of the cell multilayer/sheet (**Figure S7**). In contrast, no detachment was observed after seeding of HUVEC on an EtOH-treated NHDF layer and the HUVEC layer was maintained during the cultivation period of 14 days (Figure S7).


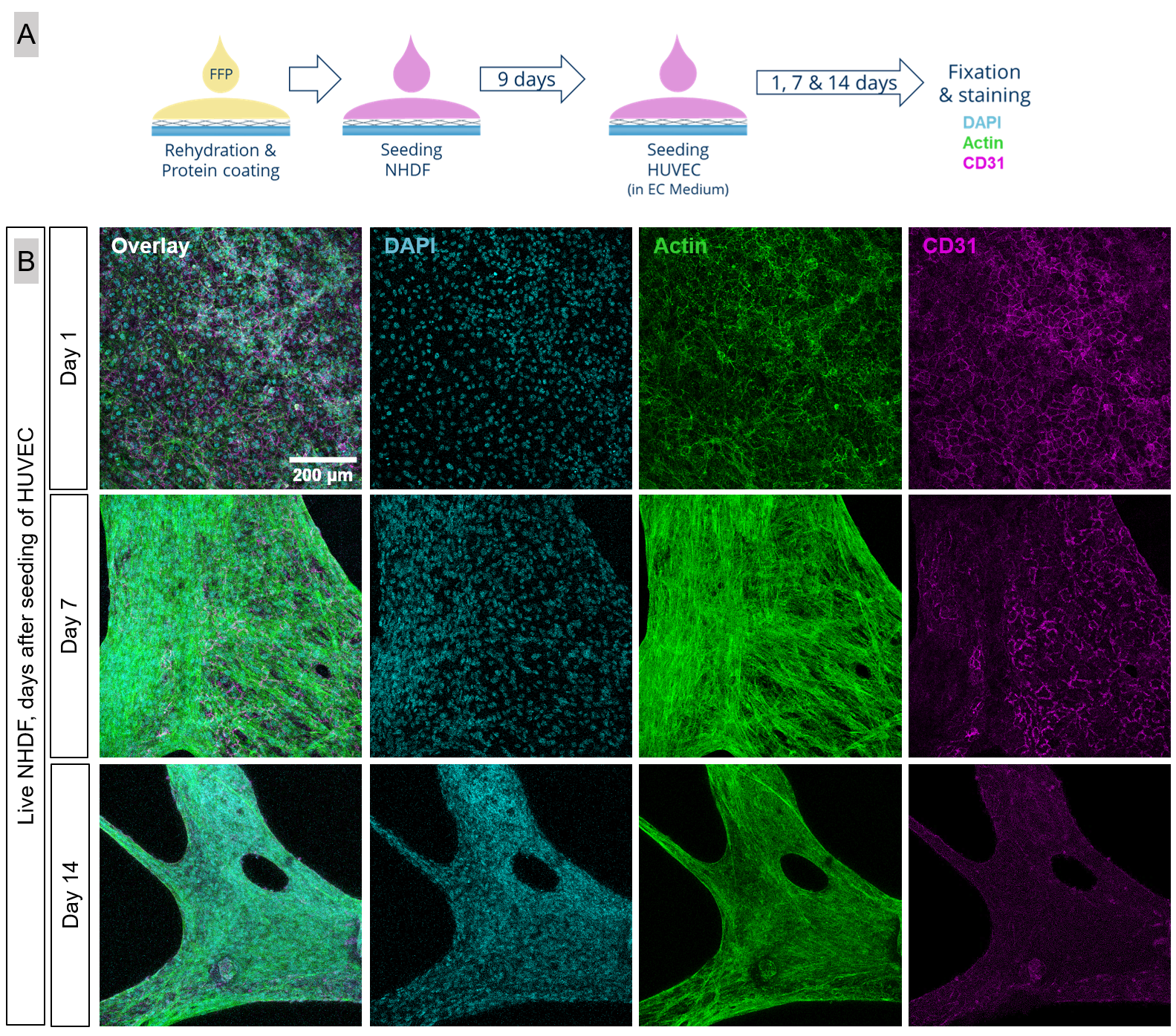


**Figure S6.** Formation of a HUVEC layer on scaffolds with a live NHDF layer (1x10^5^ NHDF seeded). The experimental setup is shown in (A); 2.3x10^5^ HUVEC/scaffold were used for seeding. After 1, 7, and 14 days of cultivation, samples were stained for cell nuclei with DAPI (blue), cytoskeletons with Phalloidin 488 (green) and the endothelial cell marker CD31 (magenta); scale bar = 200 µm for all (B).

**Figure S7.** Comparison of the HUVEC layer (2.3x10^5^ HUVEC seeded) formed on scaffolds with a live NHDF layer (A) or an EtOH-treated NHDF layer (B) after 14 days of co-cultivation. Samples were stained for cell nuclei with DAPI (blue), cytoskeletons with Phalloidin 488 (green) and the endothelial marker CD31 (magenta); scale bar = 300 µm for all. The strongly proliferating NHDF led to detachment of the multi-cell-layer, while the confluent HUVEC monolayer formed on the EtOH-treated NHDF was maintained.


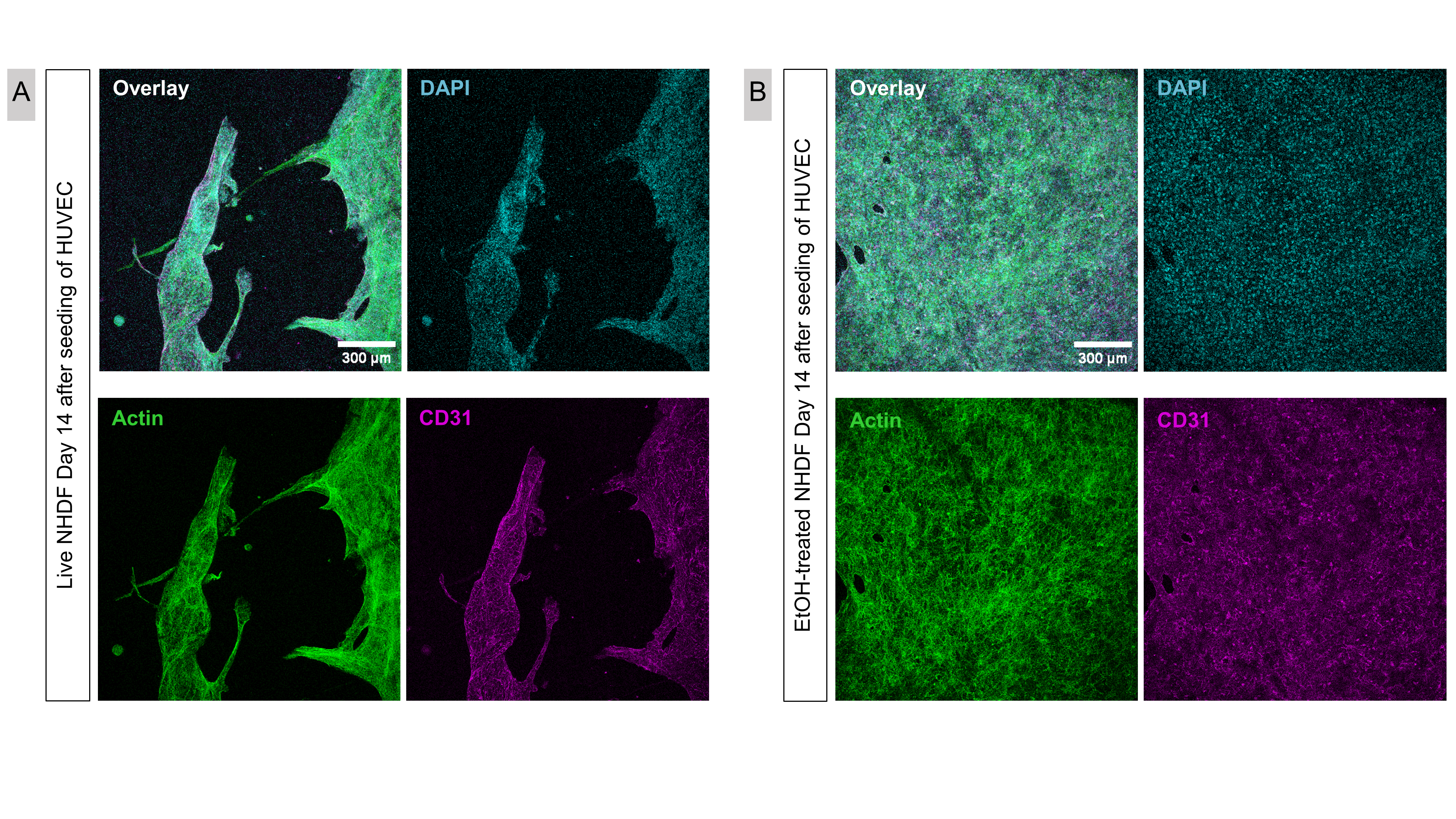


***5. Mono-cultures of smooth muscle cells***

Human umbilical vein smooth muscle cells (HUVSMC) were seeded onto an NHDF layer previously cultured on the scaffolds and treated with EtOH. The HUVSMC were cultivated in their specific smooth muscle cell growth medium for 21 days. Live cell staining at day 1, 7, 14, and 21 of cultivation revealed poor adhesion and expansion over time of the cells on the EtOH-treated NHDF layer **(Figure S8**). A direct comparison of HUVSMC attachment and development on FFP-functionalized AlgMC/PCL composite scaffolds with and without EtOH-treated NHDF layer indicated a better colonization of the scaffolds without the NHDF (**Figure S9**).


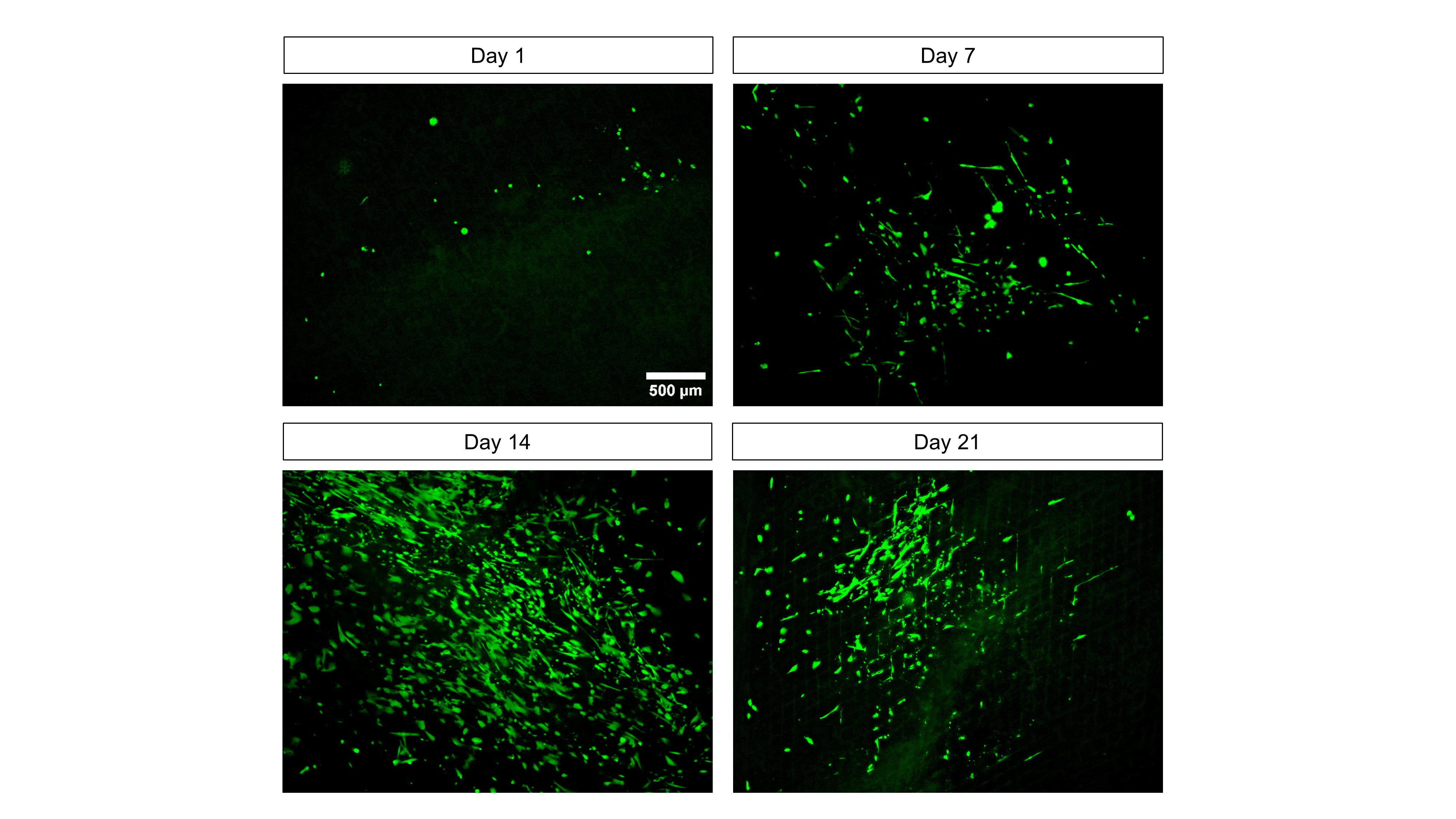


**Figure S8.** Adhesion, spreading and growth of HUVSMC seeded on FFP-functionalized AlgMC/PCL composite scaffolds with an EtOH-treated NHDF feeder layer (1x10^5^ NHDF seeded for feeder layer generation, 1.5x10^5^ HUVSMC/scaffold seeded on the feeder layer). Staining of live cells was performed at day 1, 7, 14, and 21 of cultivation; exemplary fluorescence-microscopic images are shown (scale bar = 500 µm for all).


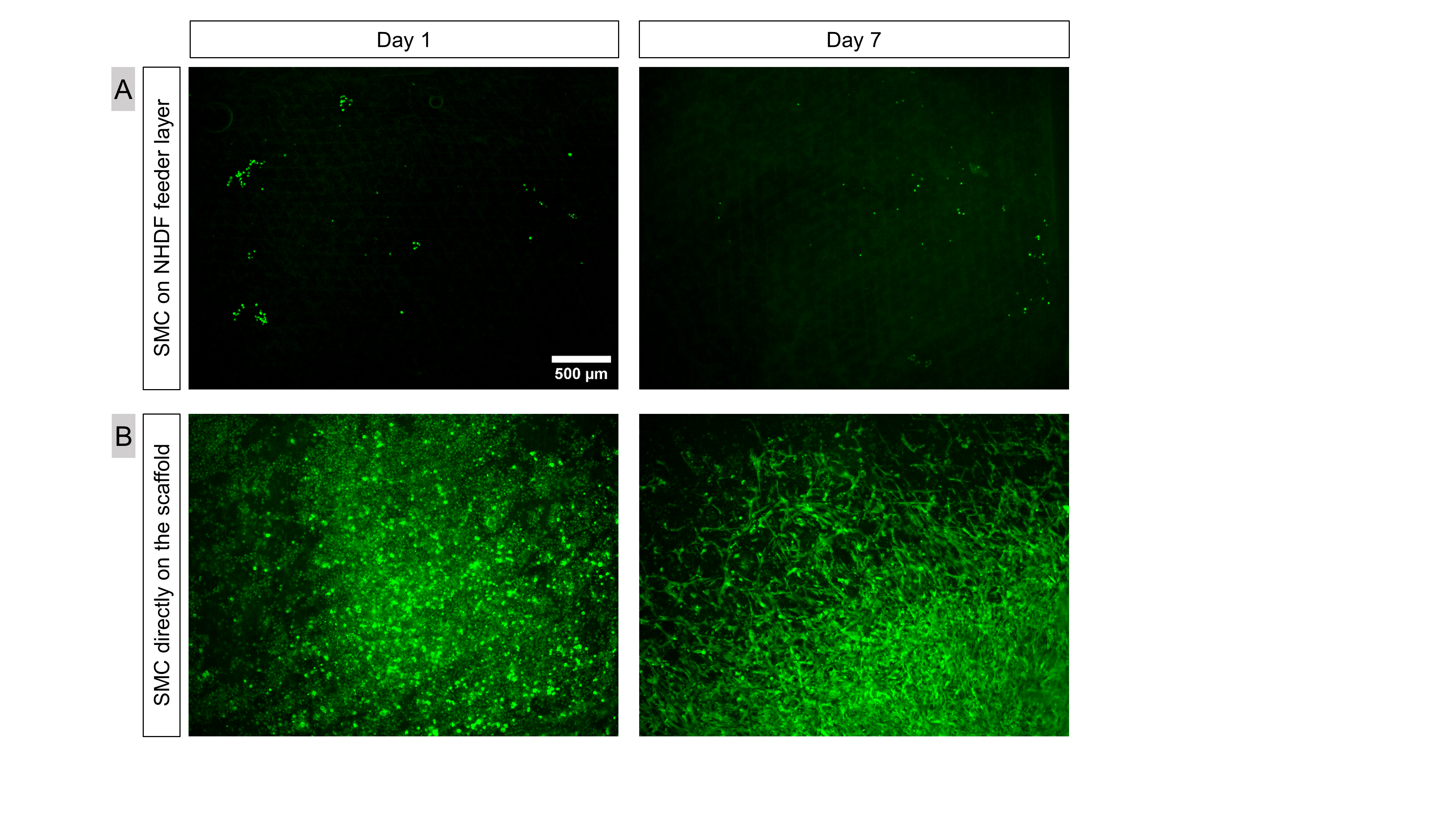


**Figure S9.** Comparison of HUVSMC adhesion, spreading and growth on FFP-functionalized AlgMC/PCL composite scaffolds with and without EtOH-treated NHDF layer. Staining of live cells was performed at day 1, and 7 after seeding (1.5x10^5^ HUVSMC/scaffold); exemplary fluorescence-microscopic images are shown (scale bar = 500 µm for all).

***6. Bilayered co-cultures of smooth muscle cells and endothelial cells***

Further experiments demonstrated that an NHDF feeder layer is not necessary to support HUVEC layer formation in a co-culture with HUVSMC, but that the seeding cell number of HUVEC is a crucial factor to achieve a fully developed endothelial monolayer. In successive seeding steps within one experiment i) a high number of HUVEC (2.3x10^5^) was seeded on HUVSMC grown on an NHDF feeder layer, ii) a low (1.2x10^5^) and a high (2x10^5^) number of HUVEC were seeded on HUVSMC grown on FFP-functionalized scaffolds without the feeder layer, and results showed that HUVEC were only able to form a confluent endothelial layer when seeded in a density of ≥ 2x10^5^ cells per scaffold (i.e., 1.54x10^4^ cells per mm^2^) (**Figure S10**).


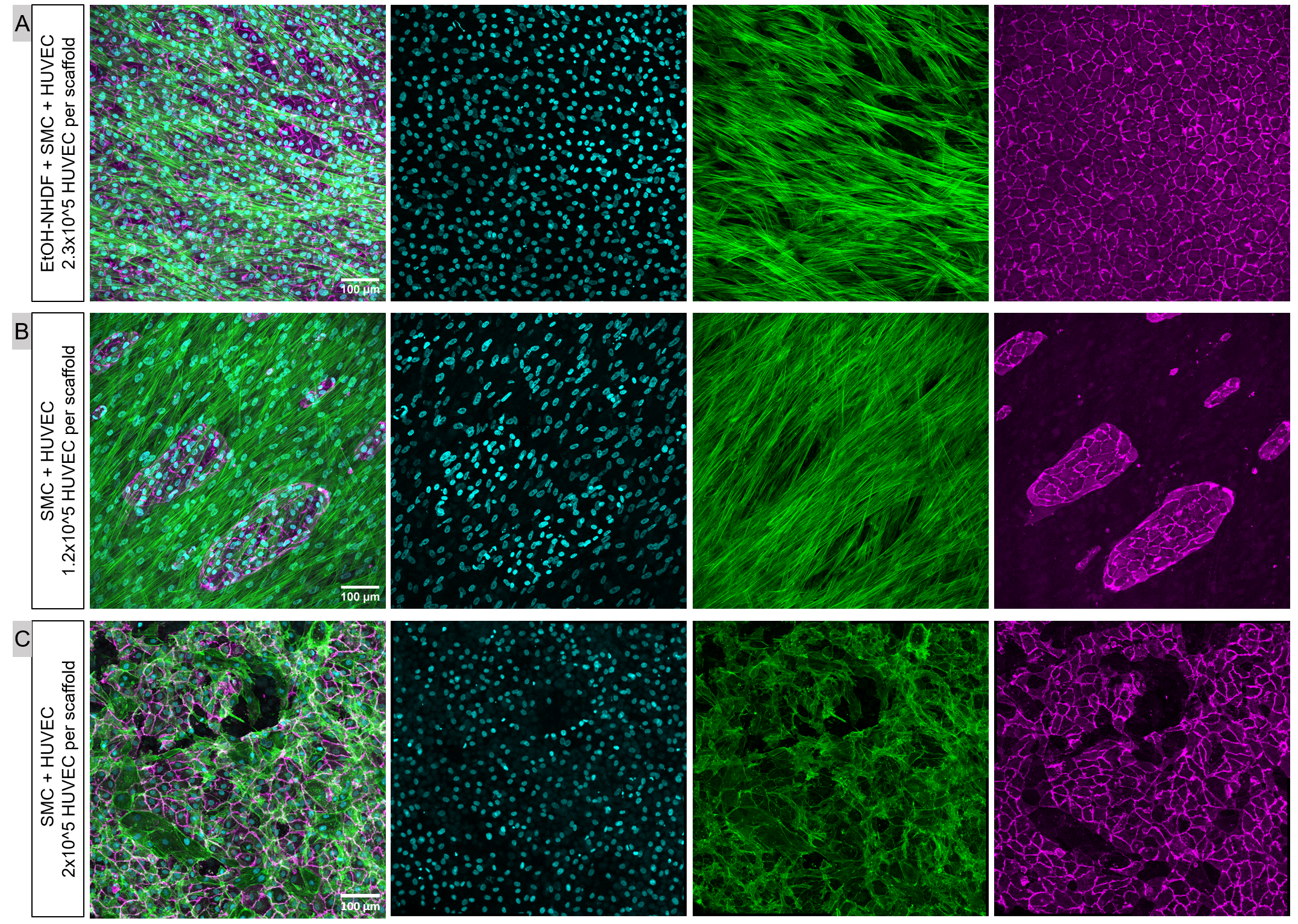


**Figure S10.** Comparison of HUVEC layer formation depending on the number of HUVEC seeded per scaffold: (A) 2.3 x10^5^, (B) 1.2 x10^5^, (C) 2x10^5^. (A) 1x10^5^ NHDF seeded for feeder layer generation, (B) and (C) without feeder layer. HUVSMC seeded were 1.5x10^5^ for all. Exemplary images taken 7 days after seeding of HUVEC. Samples were stained for cell nuclei with DAPI (blue), cytoskeletons with Phalloidin 488 (green) and the endothelial marker CD31 (magenta); scale bar = 100 µm for all. Independently of the presence of the NHDF feeder layer the co-cultures of HUVSMC and HUVEC were able to proliferate and cover the scaffolds; however if the number of HUVEC seeded was too low (B), they formed small islands within the HUVSMC layer instead of growing in a monolayer on top.
